# Biphasic Co‐Delivery of Curcumin and 5‐FU using 3D‐Printed Dissolving Microneedles for Skin Cancer Therapy

**DOI:** 10.1002/adhm.71620

**Published:** 2026-08-20

**Authors:** Rutuja N. Meshram, Dimitrios A. Lamprou

**Affiliations:** ^1^ School of Pharmacy Queen's University Belfast Belfast UK

**Keywords:** 3D printing, 5‐fluorouracil, curcumin, dissolving microneedles, skin cancer, transdermal drug delivery

## Abstract

Dissolving microneedle (MN) arrays are a minimally invasive strategy for localized transdermal chemotherapy; however, their translation is limited by drug loading, reproducible dosing, and the difficulty of incorporating chemically distinct drugs into printable matrices. Although MN‐mediated delivery of curcumin and 5‐fluorouracil (5‐FU) has previously been reported using inkjet/coating‐based approaches, those systems deposit drug formulations onto preformed solid MNs and are therefore constrained by surface coating uniformity, limited matrix integration, possible drug loss during handling, and less direct control over geometry‐dependent dose. Here, we report a distinct one‐step DLP 3D‐printing strategy in which hydrophilic 5‐FU and hydrophobic curcumin are incorporated directly into a single PEGDA/VP photocurable resin before fabrication. The optimized resin enabled high‐resolution MN arrays with CAD‐defined dimensions, sharp tips, insertion efficiency above 95%, high drug entrapment, and controlled/biphasic release behavior. Importantly, the work demonstrates that curcumin's optical absorbance can be managed through exposure optimization without compromising print fidelity, allowing dual‐drug‐loaded dissolving MNs to be manufactured directly from the therapeutic resin. These findings establish the system as a formulation‐process‐performance platform rather than a first report of curcumin/5‐FU MN co‐delivery, providing a scalable and customizable approach for localized skin‐cancer drug delivery that warrants future biological validation.

## Graphical abstract

Using pixel‐aware digital light processing, the authors created high‐resolution, dissolvable microneedles from a PEGDA/VP matrix. These microneedles encapsulate curcumin and 5‐fluorouracil and dissolve in the skin, co‐delivering drugs directly to tumours. This approach offers precise dosing, strong mechanical properties, effective penetration in skin models, and safe patient‐friendly treatment.

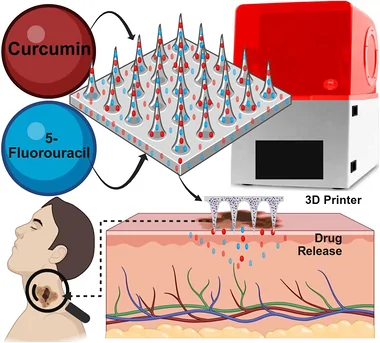

## 1 Introduction

Skin cancer, defined as the malignant transformation of cutaneous cells, is the most frequently diagnosed cancer, and its incidence continues to increase worldwide [1]. It encompasses basal cell carcinoma (BCC), squamous cell carcinoma (SCC), and melanoma. Both BCC and SCC originate from keratinocytes in the epidermis, whereas melanoma originates from melanocytes [2]. Although many non‐melanoma cancers can be cured by surgical excision, a substantial proportion of patients require supplementary interventions such as topical therapies, intralesional injections [3], photodynamic therapy [4, 5], radiotherapy [6], and systemic agents to achieve satisfactory outcomes. Topical chemotherapy is particularly appealing because it localizes drug exposure and minimizes systemic toxicity; however, its effectiveness is constrained by the stratum corneum of the skin [7]. This densely organized lipid‐protein matrix forms a formidable barrier to most small molecules, resulting in inconsistent drug delivery, prolonged treatment courses, and local irritation, which can reduce patient adherence.

MN technology has emerged as a minimally invasive strategy to overcome the stratum corneum barrier. MNs create transient microchannels that reach the viable epidermis and superficial dermis, enabling the transport of therapeutic agents without stimulating deep pain receptors [8]. Among the various designs, dissolving MNs are fabricated from water‐soluble or biodegradable polymers that encapsulate active pharmaceutical ingredients (APIs). After insertion, the needles dissolve and release the drug, eliminating sharp waste and reducing the biohazard risk. Dissolving MNs can deliver multiple agents simultaneously, and their release kinetics can be tuned by adjusting the polymer composition and MN geometry [9, 10]. By penetrating the skin to clinically relevant depths and dissolving in situ, dissolving MNs enable localized chemotherapy that restricts systemic exposure, an approach especially pertinent when systemic dosing is limited by dose‐limiting toxicities.

Additive manufacturing (AM) has revolutionized dissolving MN fabrication. Fused deposition modeling (FDM), stereolithography (SLA), DLP, and two‐photon polymerization (2PP) are key 3D‐printing (3DP) techniques used to fabricate MNs. FDM is cost‐effective but yields low resolution. Photopolymerization‐based technologies, such as SLA, DLP, and 2PP, use ultraviolet light to cure photosensitive resins and can achieve much higher resolution. 2PP offers nanometer‐scale precision but is slow and requires expensive equipment; therefore, SLA and DLP printers are more commonly used because they produce microscale features economically and rapidly. DLP is distinguished by its projection of an entire layer of light onto the resin, facilitating rapid prototyping and precise control over needle geometry, including height, base diameter, tip radius, and spacing [11, 12, 13]. By adjusting computer‐aided design (CAD) files and printing parameters, DLP can produce high‐resolution arrays with patient‐specific patch dimensions and densities. Its layer‐by‐layer photopolymerization yields mechanically robust structures that can penetrate tissues and dissolve upon hydration.

Despite these advantages, several translational challenges remain in the development of oncological dissolving MNs. Most DLP‐based MN studies have focused on the delivery of a single drug [11, 12, 14, 15, 16]. However, combination therapy is central to modern anti‐cancer management, and the co‐delivery of agents such as 5‐FU and curcumin (CUR) offers a potentially complementary therapeutic rationale [17, 18]. CUR is a natural polyphenol that can potentiate the cytotoxic effects of 5‐FU by sensitizing chemo‐resistant tumor cells and suppressing NF‐κB activation [19, 20] In a 3D alginate tumor model, co‐treatment with 5 µM CUR reduced the concentration of 5‐FU needed to inhibit proliferation of colorectal cancer cells by an order of magnitude and decreased invasion via down‐regulation of NFκB‐regulated gene products. Likewise, CUR has been shown to enhance the efficacy of multiple chemotherapeutic agents and block NFκB‐driven pathways. It is important to note that most mechanistic evidence supporting these interactions derives from colorectal and other non‑cutaneous cancer models; whether a similar interplay exists in the skin‑cancer microenvironment has not yet been demonstrated. In dermatological oncology, 5‐FU is already used clinically for actinic keratosis and superficial BCC, but it exhibits poor passive skin penetration and requires frequent, sometimes irritating applications; combining it with CUR may increase its efficacy while mitigating inflammation [21].On this basis, the combination was used as a potentially complementary therapeutic strategy, coupling by coupling a cytotoxic agent (5‐FU) with CUR, an anti‑inflammatory, antioxidant and chemo sensitizing effect. However, the simultaneous encapsulation of two chemically distinct APIs in a single photopolymerizable matrix is challenging. Achieving high loading, uniform distribution, and predictable biphasic release, an initial burst for rapid therapeutic exposure followed by sustained delivery without impairing mechanical properties or print fidelity, remains an unresolved technical barrier. Another limitation is the limited integration of the design and performance data. Many studies have reported geometric precision, mechanical strength, or release behavior individually, but few have systematically correlated resin rheology, photopolymerization chemistry, geometric fidelity, insertion capacity, drug loading uniformity, and release kinetics within a single optimized formulation. Photocurable resins, such as poly (ethylene glycol) diacrylate (PEGDA) and N‐vinylpyrrolidone (VP), are commonly used for DLP printing; however, incomplete polymerization or residual monomeric fragments can compromise biocompatibility and drug stability. Demonstrating manufacturing scalability and batch‐to‐batch reproducibility of drug loading and mechanical properties is also critical for clinical translation. Regulatory approval will require consistent fabrication, sterility, and safety.

**FIGURE 1:**
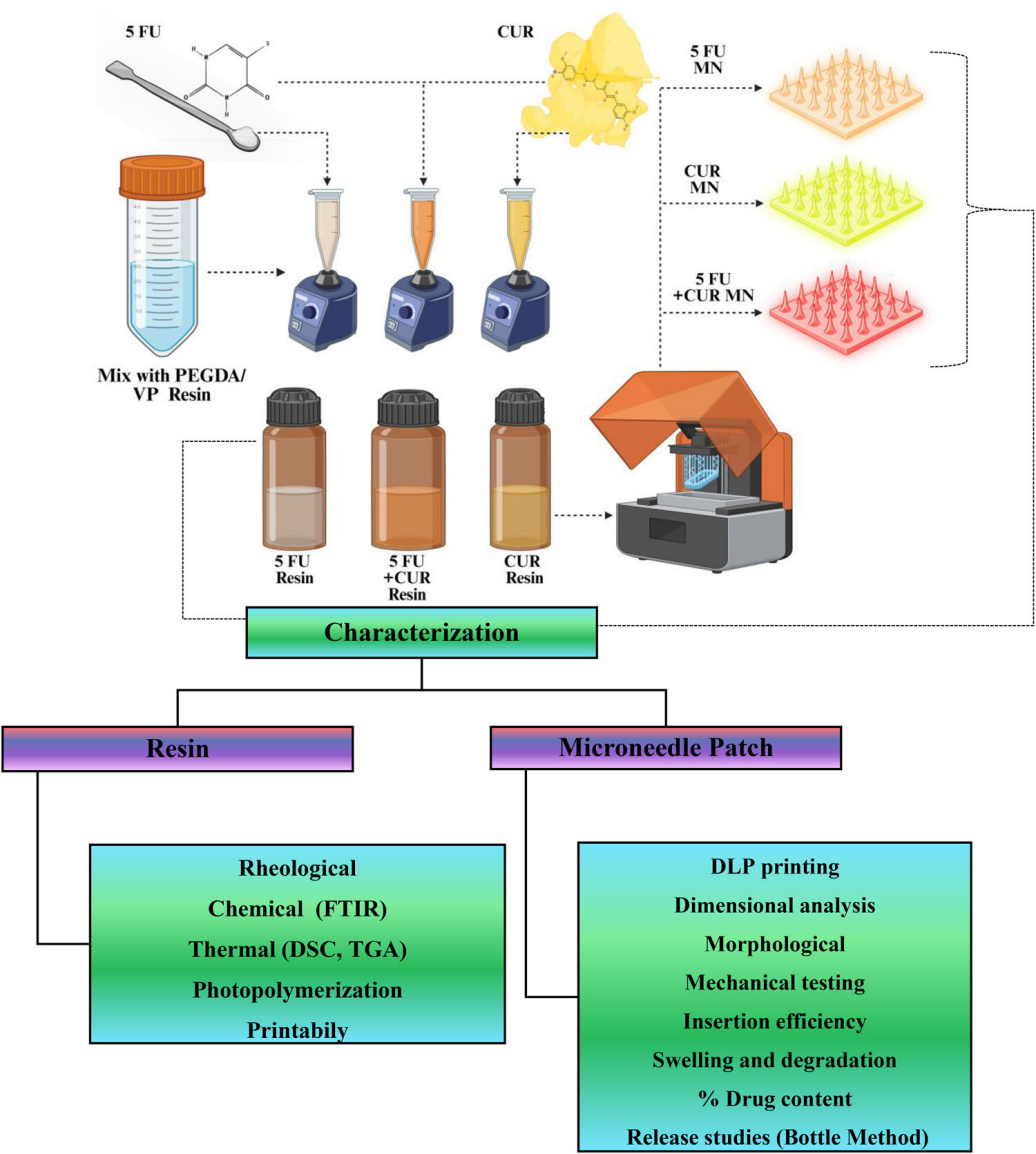
The roadmap illustrates the formulation, fabrication, and characterization of resin and MNs.

Compared with conventional topical 5‐FU creams or CUR gels, a MN platform offers several formulation‐related advantages: it can bypass the stratum corneum barrier, support localized intradermal deposition, reduce reliance on prolonged surface residence, and allow dose modulation through CAD‐controlled patch geometry. Importantly, the novelty of the present work should be considered in the context of prior MN studies, including the 2015 report by [22], in which anticancer agents, including 5‐FU and CUR, were inkjet printed as drug‐polymer coatings onto preformed transdermal MNs. That earlier study established the feasibility of coating solid MNs with anticancer agents, but the drug payload was primarily surface‐deposited and dependent on coating formulation, droplet deposition, and coating uniformity. Such coating‐based systems can be affected by limited drug loading, heterogeneous surface distribution, coating detachment or loss during handling/insertion, and restricted ability to tune dose through the internal MN matrix. By contrast, the present study uses a different material and manufacturing concept: hydrophilic 5‐FU and hydrophobic CUR are incorporated directly into a single PEGDA/VP photocurable resin and then fabricated into dissolving MN arrays by DLP 3D printing. Therefore, the advance is not simply a change in manufacturing equipment, but the integration of drug solubilization/dispersion, photopolymerization, CAD‐defined geometry, insertion performance, matrix swelling/degradation, and controlled release within one printable therapeutic platform. Recent reports of pH‐responsive, NIR‐enhanced, eutectogel and multi‐material MNs further illustrate the rapid development of multifunctional MN technologies [23, 24, 25]; however, these systems differ substantially in therapeutic payload, polymer chemistry, responsiveness, and fabrication strategy. The specific contribution of this work lies in demonstrating that a PEGDA/VP DLP‐printable matrix can accommodate two chemically distinct APIs while preserving low viscosity, print fidelity, mechanical insertion capability, high entrapment, and biphasic release. This study does not claim that CUR/5‐FU co‐delivery itself is new, nor does it establish a new biological mechanism of synergy in skin cancer. Instead, it provides a formulation‐process‐performance study of a directly printed dual‐drug dissolving MN platform and identifies the remaining need for future skin‐cancer‐specific biological validation using relevant cell, 3D tumor, and in vivo models. The schematic roadmap summarizing the overall experimental workflow for the development of resin and MN arrays is shown in Figure 1.

## 2 Results and Discussion

### 2.1 Preparation of PEGDA/VP Resins Loaded with API

Prior literature has established that PEGDA‐based photopolymer networks yield highly crosslinked and mechanically rigid structures, whereas incorporating comonomers, such as VP, enhances matrix compliance and hydrophilicity, albeit at the cost of stiffness and print precision [12, 14, 26]. VP‐rich blends often polymerize into continuous, gel‐like layers that cannot sustain high‐aspect‐ratio geometries, whereas PEGDA‐dominant systems print readily but exhibit brittle fracture under mechanical stress. Although VP polymerizes more slowly than acrylates, PEGDA‐VP copolymerization can improve the double‐bond conversion and overall cure efficiency in vat‐photopolymerization systems [12]. Indeed, prior work employing PEGDA‐VP ratios of 30:70 (w/w) demonstrated acceptable performance for DLP‐printed MNs and other drug delivery devices (DDDs) [14]. However, previous reports [12] have not systematically correlated the PEGDA/VP ratio, photoinitiator content, and API loading with print fidelity and mechanical robustness, which are crucial parameters when formulating DLP resins for dual‐drug MNs fabrication. In this work, the introduction of a photorheological‐tuned PEGDA/VP/ lithium phenyl‐2,4,6‐trimethylbenzoylphosphinate (LAP) resin system overcomes these constraints. The photoinitiator loading strongly influences polymerization kinetics and cure depth. Although LAP concentrations near 0.5% (w/w) are frequently reported in biocompatible DLP resins to ensure rapid gelation [27, 28], it was observed that such levels promote overcuring in high contrast microfeatures. CUR, a hydrophobic model drug, also acts as a photo absorber owing to its strong absorbance at ∼405 nm [29], reducing photon penetration and necessitating compensation by exposure adjustment. These competing optical effects complicate the co‐formulation with 5‐FU, a hydrophilic drug that does not absorb at printing wavelengths.

To address these challenges, systematically screened resin compositions to balance printability and post‐print integrity have already been investigated in [26]. VP‐rich blends (≥70 wt%) were polymerized into soft, low‐modulus films rather than discrete MN structures, whereas PEGDA‐rich systems (≥60 wt%) generated rigid glassy networks that fractured upon handling. The mid‐range ratios exhibited superior curing uniformity and mechanical resilience. The optimized composition, PEGDA: VP, 40:60 (w/w), achieved consistent layer adhesion, well‐formed needle geometry, and adequate toughness for post‐processing. Photoinitiator LAP 0.4 wt% was the optimal concentration: This concentration provided a reproducible cure depth of ∼25 µm per 3 s exposure in the control (blank) resin. Importantly, the optimized PEGDA/VP (40:60) base‐maintained print fidelity even in the presence of CUR optical attenuation by fine‐tuning the exposure time per layer. Regarding active incorporation, the resin tolerated up to 1.5 wt% 5‐FU and 1 wt% CUR individually without visible phase separation or loss of dimensional fidelity. For dual‐drug patches, 1.0 wt% 5‐FU + 0.5 wt% CUR (w/w) provided the most uniform curing behavior and mechanical stability. Attempts to exceed these concentrations resulted in progressive structural deformation of the printed MN geometry, including tip broadening, reduced height uniformity, and irregular base formation. At higher API loadings, residual uncured resin was frequently observed, indicating incomplete photopolymerization and compromised crosslinking efficiency. These findings collectively indicate that the reported concentrations represent the upper practical limits for API incorporation into the PEGDA‐VP photopolymerizable matrix while maintaining print integrity and reproducibility. Optical appearance correlated directly with curing kinetics; 5‐FU‐loaded resins remained optically clear and required standard exposure, whereas CUR‐containing systems exhibited progressive yellowing and required incremental increases in exposure to offset light absorption.

### 2.2 Flow Behavior and Rheological Analysis of Resins

Rheological control plays a decisive role in vat photopolymerization processes, such as SLA and DLP. The resin viscosity determines the ease of flow and uniformity of layer re‐coating between successive light exposures. Excessively viscous systems (above ∼10^2^–10^3^ mPa·s) impede resin flow and can cause incomplete layer replenishment or air entrapment, whereas very low‐viscosity formulations flow readily but may suffer from surface instabilities and sedimentation of additives [30, 31]. During DLP printing, the resin typically experiences transient shear rates of approximately 80–100 s^−^^1^ as the build platform separates and recontacts the vat; under these conditions, maintaining a stable viscosity is essential to ensure uniform deposition and reproducible polymerization. The optimized PEGDA/VP (40:60 w/w) photo resin developed in a previous study [26], exhibited a stable viscosity of 0.1 Pa·s (10 mPa·s) across relevant shear rates, remaining well below the critical upper threshold for DLP processing. This low and consistent viscosity enabled smooth, defect‐free recoating, and rapid self‐levelling, directly contributing to the high geometric fidelity observed in the printed MN arrays. Increasing the PEGDA fraction resulted in a monotonic increase in viscosity, confirming its dominant influence on the network hydrodynamic behavior.

**FIGURE 2:**
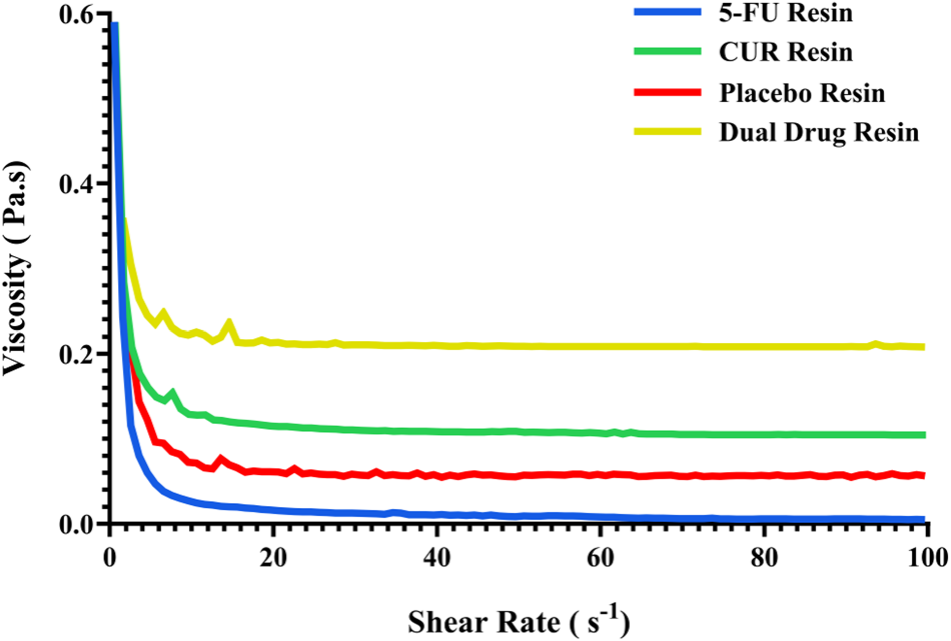
Viscosities of formulations under continuous shear rate sweep (*n* = 3).

The incorporation of CUR and 5‐FU had a negligible impact on the bulk viscosity, indicating that both drug‐loaded resins retained the favorable flow properties of the base formulation. Both formulations exhibited mild shear‐thinning behavior at low shear rates, transitioning to a nearly Newtonian plateau at higher shear rates. Specifically, the viscosity of the CUR‐loaded resin decreased sharply with increasing shear and stabilized near ∼15 s^−^^1^, whereas the 5‐FU formulation reached a plateau at approximately ∼10 s^−^^1^ (Figure 2). Beyond these points, the viscosity remained constant across the operational shear range. This rheological profile, characterized by moderate shear thinning followed by a high shear plateau, is advantageous for DLP printing. Under quiescent conditions, the resin possesses sufficient viscosity to prevent settling or premature spreading, whereas during recoating, shear‐induced thinning promotes rapid and uniform flow. Once the shear ceases, viscosity recovery preserves the layer uniformity and print resolution. This balance between flowability and stability enables precise layer stacking, minimizes interfacial defects, and ensures reproducible photopolymerization kinetics. In contrast to conventional PEGDA‐dominant DLP formulations, which often rely on external diluents or reactive solvents to adjust viscosity, this PEGDA/VP blend intrinsically achieves an optimal rheological window through its compositional design. The resulting low‐viscosity and shear‐responsive behavior provides a robust foundation for printing both drug‐loaded MN, demonstrating that dual‐drug incorporation does not compromise printability or layer fidelity.

### 2.3 DLP Fabrication of Single and Dual Drug MN Arrays

Earlier investigations into DLP 3DP have shown that the build fidelity is strongly governed by the optical dose per layer (irradiance × exposure time) and light‐transmission behavior of the resin [32]. Transparent formulations polymerize at lower doses, whereas photo‐absorbing or light‐scattering systems require longer exposure times to achieve comparable cure depths. However, excessive irradiation can trigger lateral or axial “bleed,” overcuring that blunts MNs tips and broadens the sidewalls. Most reported DLP MN protocols employ irradiances in the range of ∼22–27 mW cm^−^^2^, with exposure times adjusted according to resin composition and layer thickness [12, 26]. These principles also predict that chromophore drug molecules such as CUR, which absorb near 405 nm, attenuate the projected light and thus demand higher optical doses to achieve complete polymerization.

In this study, pixel‐aware exposure optimization was implemented to compensate for the specific absorbance characteristics of each drug‐loaded resin. Two CAD‐defined array geometries were designed to evaluate both fabrication reproducibility and drug‐dependent optical effects: Design A (placebo or single drug) consisted of a 6 × 6 array (16 needles) on a 6 × 6 × 1.5 mm patch. Design B (dual drug) featured a 10 × 10 array (64 needles) on a 10 × 10 × 1.5 mm patch. Both maintained an identical MNs height (1.5 mm) to isolate the effect of the array footprint on print integrity and potential drug payload. Printing was performed at an irradiance of 26.61 mW cm^−^^2^ with a nominal layer thickness of 25 µm. For the optically clear placebo (PEGDA: VP = 40:60 w/w, 0.4% LAP) and 5‐FU resins, an exposure time of 3 s per layer produced fully formed needles with sharp tips, smooth sidewalls, and no delamination. Measured heights matched CAD specifications. In contrast, the CUR‐loaded resin, which was visually yellow and partially opaque, showed an incomplete height and rounded tips at the same exposure, indicating under‐curing due to light attenuation. Incremental optimization identified 8 s per layer as the minimum exposure to restore full height and definition. For the dual‐drug resin (1.0 wt% 5‐FU + 0.5 wt% CUR), an intermediate 5 s per layer exposure yielded uniform, defect‐free needles across the 10 × 10 array, demonstrating that resin composition, rather than array size, dictated light‐dose requirements. Notably, even under prolonged exposure to the CUR formulation, no feature broadening or base overcuring occurred. This observation suggests that CUR intrinsic optical absorbance imposes a self‐limiting effect on light propagation, confining polymerization within each projected layer. Such auto‐modulated curing behavior mitigates the need for external photo absorbers or mechanical masks typically used to control over‐exposure in DLP printing. The optimized fabrication parameters, 26.61 mW cm^−^^2^ irradiance with 3 s (placebo/5‐FU), 5 s (dual‐drug), and 8 s (CUR), consistently produced high‐fidelity MN arrays (16‐ and 64‐needle designs) with smooth morphology and accurate dimensions. This reproducibility across formulations confirms that calibrating the exposure time to the optical properties of each resin provides a robust, generalizable approach for DLP printing of hydrophilic and hydrophobic drug‐loaded MNs on a unified material platform. Unlike earlier MN fabrication studies, which relied on single‐resin or dye‐modified systems, this work demonstrates that the inherent absorbance of active molecules can be harnessed as a functional tool for fine‐tuning polymerization.

This direct‐printing strategy also differentiates the present platform from inkjet‐coated or cast MN systems [22]. In coating‐based approaches, drug deposition occurs after the MN structure has been produced, so dose depends on droplet deposition, drying behavior, coating adhesion, and the accessible external surface area. In the present DLP system, the drug‐loaded resin itself becomes the MN matrix, meaning that geometry, payload, swelling, degradation, and release are designed together rather than sequentially. This is particularly relevant for the co‐formulation of 5‐FU and CUR because the two drugs differ markedly in aqueous solubility and optical behavior. The ability to maintain printability and dimensional fidelity while compensating for CUR‐mediated light attenuation provides a technological advantage beyond a simple change in fabrication technique. Furthermore, CAD‐based control of patch size, needle number, needle height, spacing, and array footprint enables dose adjustment without changing the basic formulation chemistry, which is difficult to achieve reproducibly with coating‐only methods.

### 2.4 Spectroscopic Confirmation of Drug Polymer Network

**FIGURE 3:**
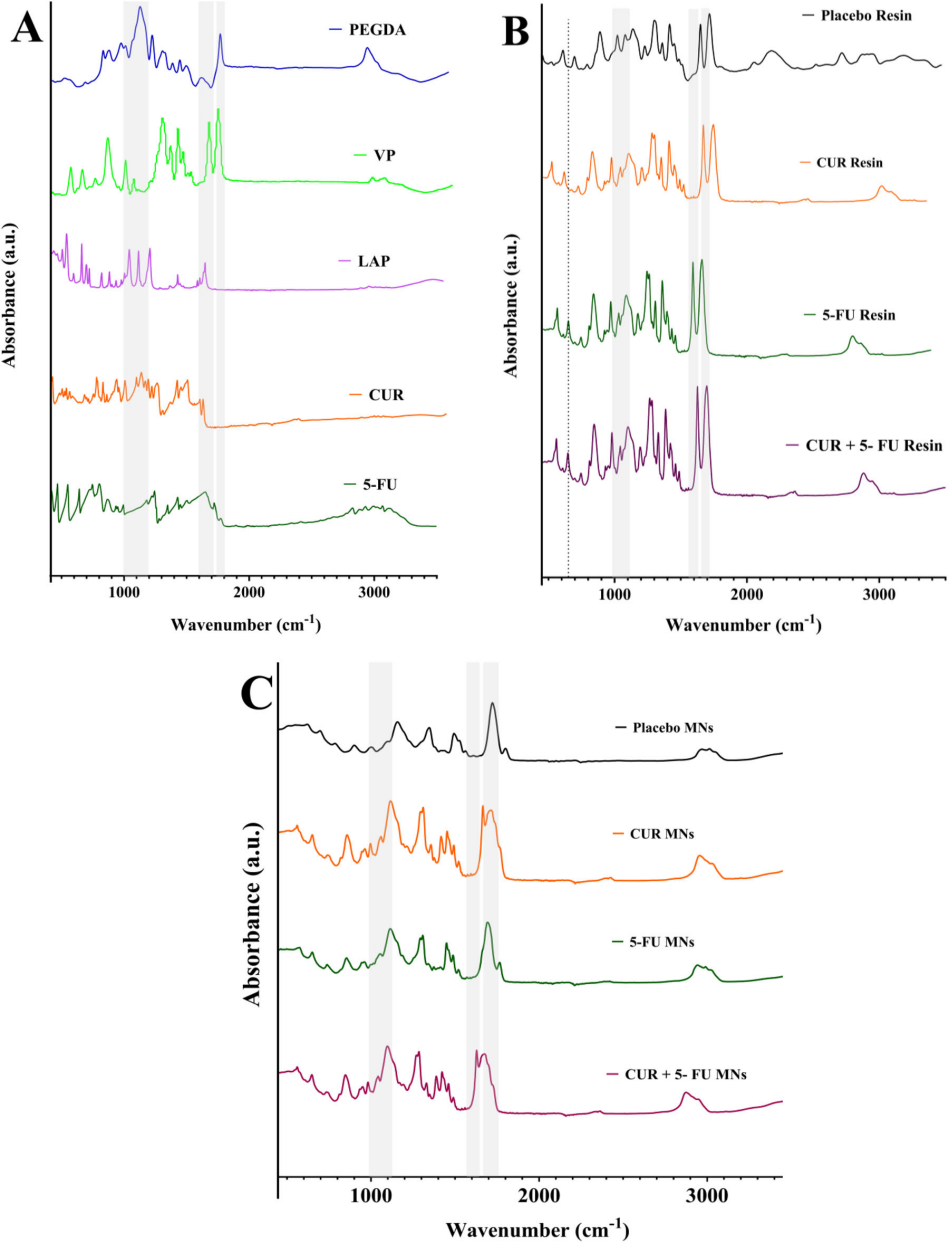
ATR‐FTIR characterization of materials, resins, and MN matrices. (A) Spectra of neat components (PEGDA, VP, LAP, CUR, and 5‐FU) with characteristic bands. (B) Uncured resins (placebo, CUR‐loaded, and 5‐FU‐loaded. (C) Cured MN formulations (placebo, CUR, 5‐FU, and CUR+5‐FU). Light grey shaded regions indicate the characteristic C–O–C stretching region (∼1000‐1150 cm^−^^1^), C = C stretching region (∼1620–1640 cm^−^^1^), and C = O stretching region (∼1700‐1725 cm^−^^1^). The reduction or disappearance of the C = C band after curing is consistent with PEGDA/VP photopolymerization. Representative spectra are shown (*n* ≥3).

Attenuated total reflectance‐fourier transform infrared (ATR‐FTIR) spectroscopy was used to assess photo‐polymerization and confirm drug incorporation within the PEGDA/VP MNs matrices. Spectra of the neat components (PEGDA, VP, LAP, CUR, and 5‐FU) reported in Figure 3A, the uncured resin in Figure 3B, and UV‐cured MN patches (placebo and API‐loaded) in Figure 3C were compared to monitor functional group conversion and interactions among resin constituents along with characteristic peaks in Table 1. The spectral interpretation presented here is based on previously published FTIR analyses of PEGDA/VP‐based photopolymer networks [26], but the description has been independently rewritten and adapted for the present formulation. The uncured PEGDA/VP resin exhibited characteristic PEGDA methylene stretching vibrations (∼2885–2895 cm^−^^1^), acrylate carbonyl (C = O) stretching near 1715–1725 cm^−^^1^, and distinct vinyl (C = C) absorptions around 1620–1635 cm^−^^1^, along with C–O–C ether linkages (∼1000‐1120 cm^−^^1^). Features attributable to the VP monomer, including lactam C = O (∼1670 cm^−^^1^) and C–N stretching (∼1280 cm^−^^1^), were also present. After DLP curing, the vinyl‐related peaks (≈1620‐1640 cm^−^^1^ and out‐of‐plane = C–H bending at ∼810‐830 cm^−^^1^) disappeared or markedly decreased, confirming extensive double‐bond conversion and network formation. Concurrent enhancement of the polymer‐backbone bands, especially the ester C = O (∼1720 cm^−^^1^) and ether C–O–C (∼1000‐1100 cm^−^^1^) stretches, indicated the establishment of an acrylate‐lactam copolymer framework. Broadening within 1660–1740 cm^−^^1^ is consistent with hydrogen‐bonded carbonyl species within the crosslinked PEGDA‐VP matrix, with the absence of discernible LAP peaks further supporting complete photopolymerization and negligible residual initiator as both the. The drug‐loaded formulations displayed spectral features, confirming successful encapsulation without chemical degradation. CUR exhibited diagnostic phenolic O–H stretching (3200–3550 cm^−^^1^), mixed C = C/C = O vibration (∼1620‐1630 cm^−^^1^), aromatic C = C (∼1600 cm^−^^1^), and enolic C–O/C–O–C absorptions (∼1270/∼1020 cm^−^^1^).

**TABLE 1:** FTIR spectroscopy: Peak wavelengths and functional groups.

Material	Wavelength (cm^−^^1^)	Functional Group
VP	1750; 1634; 1422‐1382 [33]; 845	Lactam C = O; C = C; CH bending; = C–H
PEGDA	2889; 1721 [34]; 1623; 1110; 960/843	CH_2_; acrylate C = O; vinyl C = C; C–O–C; = CH out‐of‐plane
CUR (neat)	3293/3506; 1626; 1601; 1508; 1272; 1023 [35, 36].	O–H; mixed C = C/C = O; aryl C = C; C = O; enolic C–O; C–O–C
5‐FU (neat)	3000‐2900; 1660‐1429; 1348; 1246/1180 [37, 38].	C–H; ring C = N/C = C; pyrimidine mode; C–N/C–O
Placebo MNs (cured PEGDA/VP)	1660‐1740 [12, 39]; 1000–1040; (no 1620–1640 vinyl) [26].	Carbonyl envelope; C–O–C; vinyl consumption
CUR‐MNs	∼3313 [40]; ∼1625; ∼1500	O–H; CUR diagnostic; CUR diagnostic
5‐FU‐MNs	1000‐1040; 1190 [1]	C–O–C; C–O/C–N (overlapping polymer)
Combination MNs (CUR+5‐FU)	∼1625; ∼1500; 1660–1740; (no 1620–640 vinyl)	CUR diagnostics; carbonyl envelope; polymerized network

In CUR‐loaded MNs, characteristic peaks at ∼1625 and ∼1500 cm^−^^1^ remained visible against the polymer background, accompanied by minor carbonyl broadening, consistent with non‐covalent hydrogen‐bond interactions between CUR and the PEGDA/VP matrix rather than covalent linkage. 5‐FU presented C–H stretches (∼2900–3000 cm^−^^1^), ring C = N/C = C absorptions (1430‐1660 cm^−^^1^), and C–O/C–N stretching (∼1180‐1250 cm^−^^1^). In the 5‐FU‐loaded MNs, these signals were largely masked by the dominant polymer bands, although faint shoulders near 1200 cm^−^^1^ indicated physical entrapment of the drug within the cured network. The dual‐drug (CUR + 5‐FU) system reproduced the CUR diagnostic peaks superimposed on the polymer spectrum without the appearance of new absorptions or residual vinyl signatures. The absence of additional peaks suggests that both drugs were incorporated through physical confinement and intermolecular interactions rather than chemical bonding while maintaining complete polymerization. Collectively, these findings verified the formation of a chemically stable PEGDA/VP crosslinked network and the successful encapsulation of both CUR and 5‐FU. The absence of unreacted monomeric bands and preservation of drug‐specific vibrations confirmed that the dual‐drug resin formulation enabled efficient curing and molecular‐level compatibility between the hydrophilic (5‐FU) and hydrophobic (CUR) components. Since the spectra of all cured MN formulations showed the complete disappearance of characteristic C = C stretching bands associated with unreacted acrylate monomers, confirming that the printed structures were fully polymerized and free from residual monomeric species. This outcome demonstrates that the optimized printing and post‐processing conditions ensured complete crosslinking across placebo, single‐drug, and dual‐drug MN systems. These findings are consistent with prior analysis of the PEGDA‐VP MN platform, where ^1H‐NMR evaluation in earlier work confirmed efficient conversion of monomers within the same resin system [26], further supporting the robustness of the current fabrication approach. Collectively, the FTIR results reinforce that the manufactured MNs meet the chemical integrity requirements necessary for safe and reliable transdermal delivery.

### 2.5 Thermogravimetric Analysis of Resin and MNs

**FIGURE 4:**
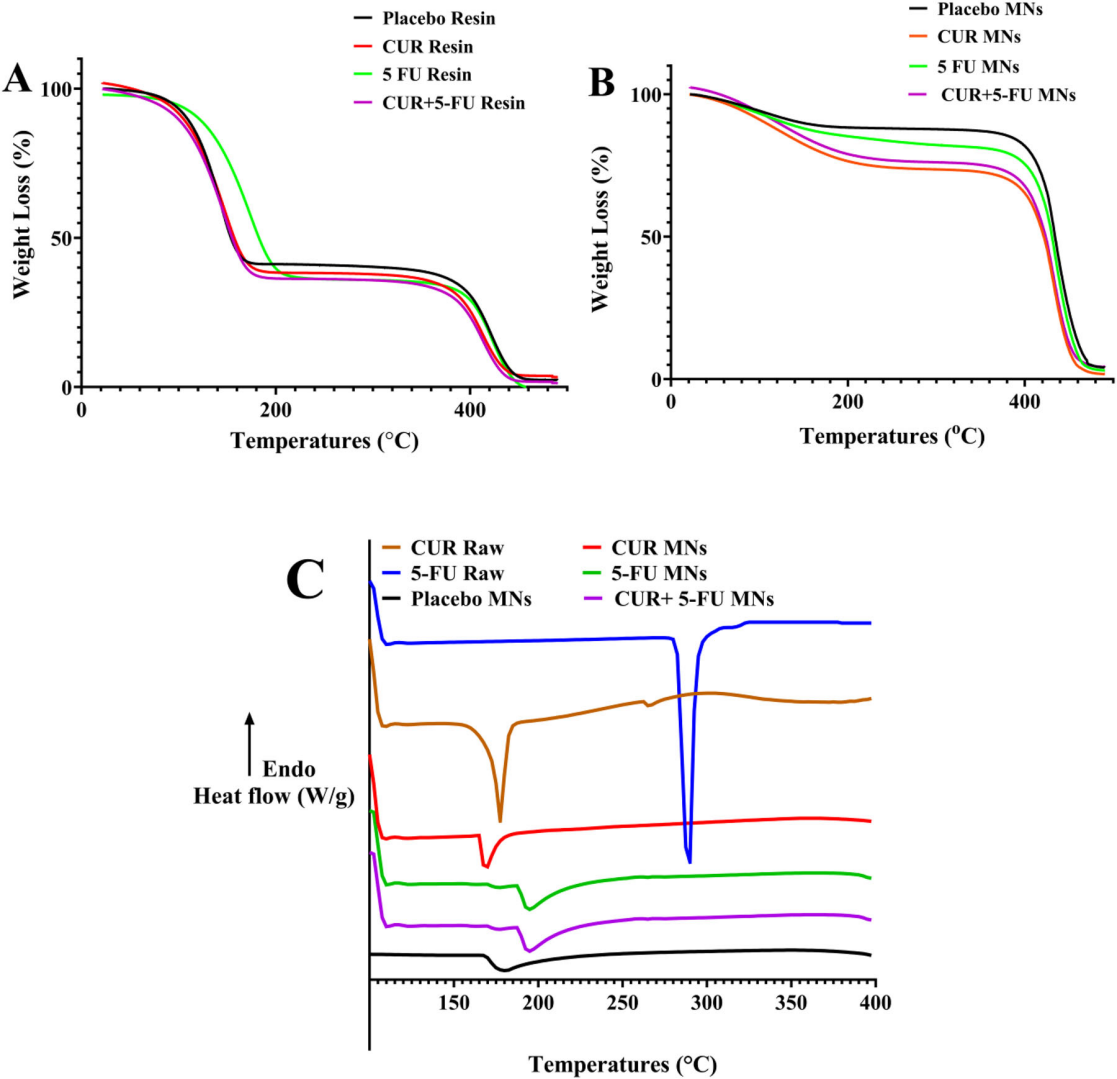
TGA and DSC analyses of PEGDA/VP‐based resins and MN matrices. (A) Uncured resins (placebo and drug‐loaded) display two‐step mass loss from the VP‐ and PEGDA‐rich fractions. (B) Cured MNs show a single high‐temperature degradation event. Minor variations in degradation profiles between formulations indicate the influence of drug incorporation on thermal stability, while overall trends confirm the formation of a thermally stable polymeric matrix. (C) DSC thermograms of APIs and MNs (*n* = 3 independent runs).

Thermogravimetric analysis (TGA) was conducted on the neat constituents (PEGDA, VP, CUR, and 5‐FU), uncured PEGDA/VP resin, and cured MN matrices (placebo and drug‐loaded) under the conditions described in the Methods. The uncured PEGDA/VP resin displayed a two‐step mass‐loss profile, preceded by a minor weight loss below ∼120 °C, attributable to residual moisture and volatiles. The first major degradation event, initiated just above 100 °C, is consistent with volatilization of the VP‐rich fraction, whereas the second higher‐temperature step (>400 °C) corresponds to PEGDA‐dominated scission of the acrylate network [26]. This dual‐event pattern reflects a partially reacted blend in which PEGDA and VP retain distinct thermal transitions. In contrast, all cured MN matrices (placebo, CUR‐MNs, 5‐FU‐MNs, and CUR + 5‐FU MNs) exhibited a single dominant degradation event at elevated temperatures (>400 °C), with the lower‐temperature VP‐related step largely suppressed. The consolidation into a single high‐temperature mass‐loss event, accompanied by an upward shift in both *T*_onset_ and *T*_max_, indicates the formation of a copolymerized PEGDA/VP network that limits volatilization and enhances the overall thermal stability. This behavior aligns with the FTIR evidence for extensive vinyl conversion and network crosslinking. Importantly, the incorporation of CUR and 5‐FU at the studied concentrations did not introduce additional low‐temperature shoulders or depress the decomposition thresholds. The thermograms of the CUR‐ and 5‐FU‐loaded MNs closely followed those of the placebo MN, and the dual‐drug formulation displayed an identical single‐step degradation pattern. Similarly, an initial mass loss of up to ∼10% in the printed arrays is attributed to the removal of absorbed moisture and residual low‐molecular‐weight species within the hydrophilic PEGDA/VP network, a trend also reflected in [26] studies. These results confirmed that both APIs were physically entrapped within the crosslinked polymer matrix without appreciable plasticization or phase separation detectable by TGA. Representative thermograms are shown in Figure 4, illustrating (A) uncured PEGDA/VP resins (placebo, CUR, and 5‐FU‐loaded) with their characteristic two‐step decomposition, and (B) cured MNs (placebo and API‐loaded) exhibiting a single, high‐temperature degradation event consistent with complete polymerization.

### 2.6 Thermal Transitions of MNs by DSC

Differential scanning calorimetry (DSC) was employed to investigate the physical state of the drugs and polymer‐drug interactions within the cured PEGDA/VP/API MN matrices (Figure 4C). The thermogram of raw 5‐FU exhibited a sharp endothermic peak at ∼283 °C, corresponding to its crystalline melting point, which was also retained in the physical mixture, confirming the persistence of the crystalline phase ex situ. In contrast, this characteristic melting peak was absent in the 5‐FU‐loaded MNs, indicating a loss of crystallinity and suggesting molecular dispersion or confinement of 5‐FU within the crosslinked PEGDA/VP network. A broad endothermic transition centered around ∼170–200 °C was observed in both placebo and drug‐loaded MNs, which can be attributed to polymer network relaxation rather than crystalline melting, as also reported in similar PEGDA‐based systems [12]. Notably, a slight shift and increased intensity of this transition in the 5‐FU‐loaded MNs suggest interactions between the incorporated drug and the polymer matrix. For CUR, the raw material displayed a distinct melting endotherm at ∼183 °C, consistent with its crystalline nature. This peak was absent in the CUR‐loaded MNs, where only a weak and broadened transition in the 160–190 °C region was observed, indicating amorphization and possible non‐covalent interactions, such as hydrogen bonding, with the PEGDA/VP network. The dual‐drug (CUR + 5‐FU) MNs exhibited a single broad transition within the same temperature range, without any detectable melting peaks of either API, suggesting a matrix‐dominated thermal behavior. Overall, the disappearance of characteristic crystalline melting events for both drugs, along with minor shifts in the polymer relaxation region, indicates good drug‐polymer miscibility and supports the formation of a predominantly amorphous system, which is favorable for uniform drug release, mechanical integrity, and formulation stability in DLP‐printed MNs.

### 2.7 Microscopic Morphology and Dimensional Fidelity of MNs

**FIGURE 5:**
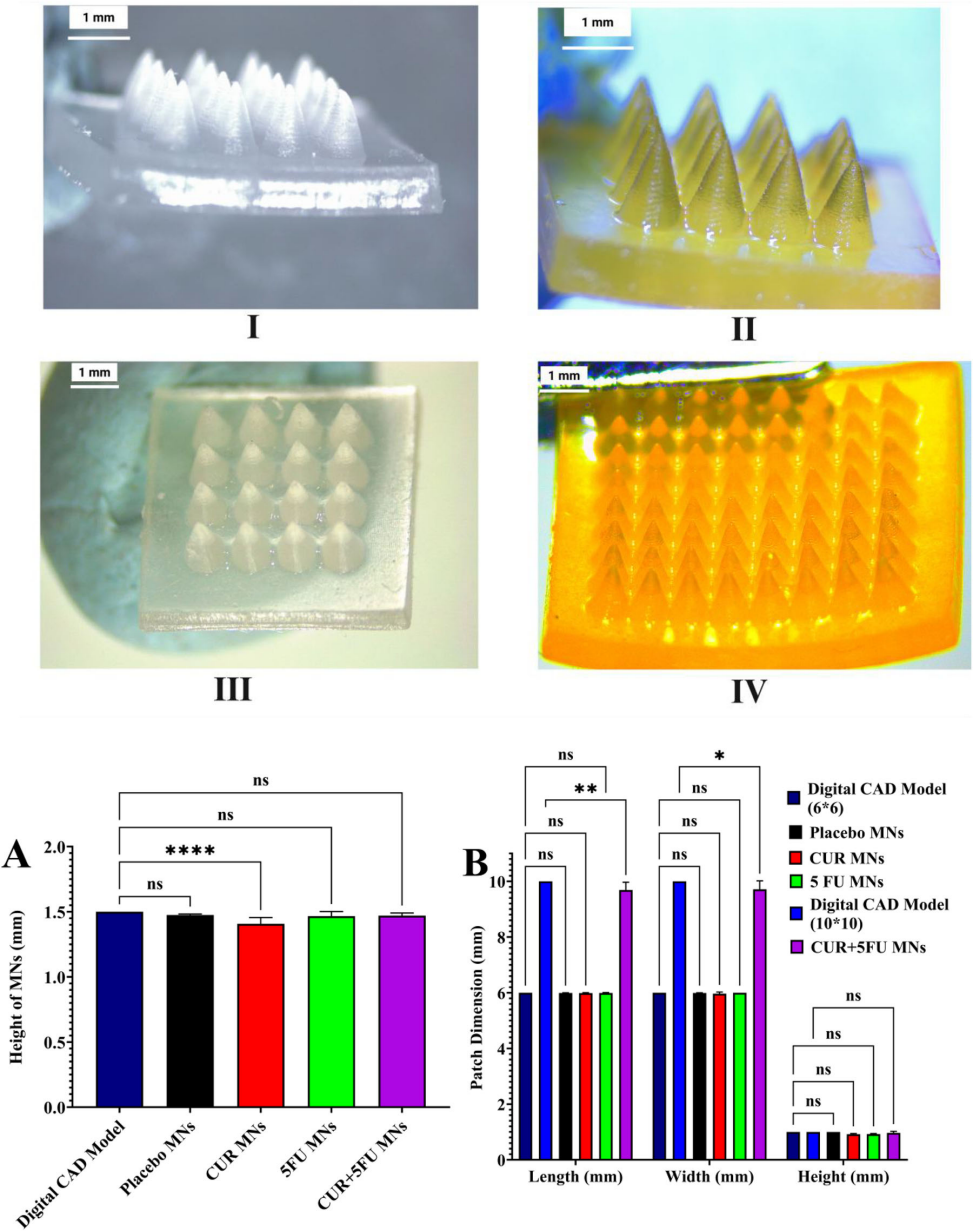
Dimensional characterization of MNs patches. Optical micrographs of MN: (I) placebo, (II) CUR, (III) 5‐FU, and (IV) CUR+5‐FU. Graphical illustration of A (*p* **** <0.0001) patch length, patch width, and B (*p* ** <0.0065, * <0.0146) needle height (mm) across formulations (placebo, 5‐FU, CUR, and CUR+5‐FU). Data are shown as mean ± SD (*n* ≥3).

MN morphology is a critical parameter governing insertion efficiency, drug delivery performance, and overall clinical functionality. In this study, the geometric accuracy and surface characteristics of the printed PEGDA/VP MN arrays were evaluated using optical microscopy (Figure 5I, II, III, IV) and scanning electron microscopy (SEM). The dimensional data were compared with CAD specifications to assess the precision of the DLP fabrication process. Optical microscopy revealed that both placebo and API‐loaded MN arrays retained exceptional fidelity to their design models. The measured dimensions of the placebo MNs were 99.35% of the intended CAD values, whereas those of the 5‐FU‐ and CUR‐loaded MNs were 98.0% and 97.33%, respectively. The dual‐drug CUR + 5‐FU MNs exhibited a slightly lower but still excellent fidelity of 95.33% (Table 2). A comparative plot of MN height and patch dimensions (Figure 5A, B) further illustrated consistency across formulations, showing minimal variation between replicates. The base patch dimensions also closely matched their CAD blueprints (6 × 6 × 1.5 mm for single‐drug and placebo MNs, 10 × 10 × 1.5 mm for dual–drug MNs), confirming precise reproduction of both individual needle and patch‐level geometry. The selected needle height of 1.5 mm was based on the anatomical characteristics of human and cancerous skin. Non‐melanoma skin lesions, including superficial basal‐cell and squamous‐cell carcinomas, typically localize within the epidermis and upper dermis and are generally reported to extend no deeper than approximately 1.0–1.2 mm, with published median invasion depths remaining well below 1 mm in most cases, as demonstrated by [41]. MNs of ∼1.5 mm, therefore, penetrate through the stratum corneum and viable epidermis while avoiding contact with pain receptors and vascular networks in the lower dermis, ensuring efficient intradermal drug deposition without bleeding. This geometry is thus optimal for localized chemotherapeutic delivery to shallow tumor regions while maintaining minimal invasiveness. Microscopic inspection confirmed that all MNs exhibited sharp tips, uniform heights, and well‐defined base diameters across the formulations. The incorporation of 5‐FU and CUR did not deform or blunt the needle geometry, underscoring the mechanical integrity of the PEGDA/VP resin system. Maintaining geometric fidelity ensures proper skin penetration and reproducible dosing performance; even minor tip deviations have been reported to reduce the insertion depth and compromise the drug delivery efficiency [42]. The consistency observed here supports reliable transdermal performance across both single‐ and dual‐drug patches.

**TABLE 2:** Practical values of MN patch dimensions and heights by optical microscopy.

Dimensions	Patch Length (mm)	Patch Width (mm)	Patch Height (mm)	Needle Height (mm)
CAD 6*6	6.00	6.00	1.00	1.50
Placebo MNs	5.88 ± 0.17	6.00 ± 0.19	1.0 ± 0.06	1.49 ± 0.15
5 FU MNs	5.54 ± 0.33	6.00 ± 0.35	1.0 ± 0.08	1. 47 ± 0.13
CUR MNs	6.0 ± 0.23	6.10 ± 0.12	1.0 ± 0.02	1.46 ± 0.04
CAD 10*10	10.00	10.00	1.00	1.50
CUR+5FU MNs	9.94 ± 0.97	10 ± 1.0	0.97 ± 0.07	1.43 ± 0.11

**FIGURE 6:**
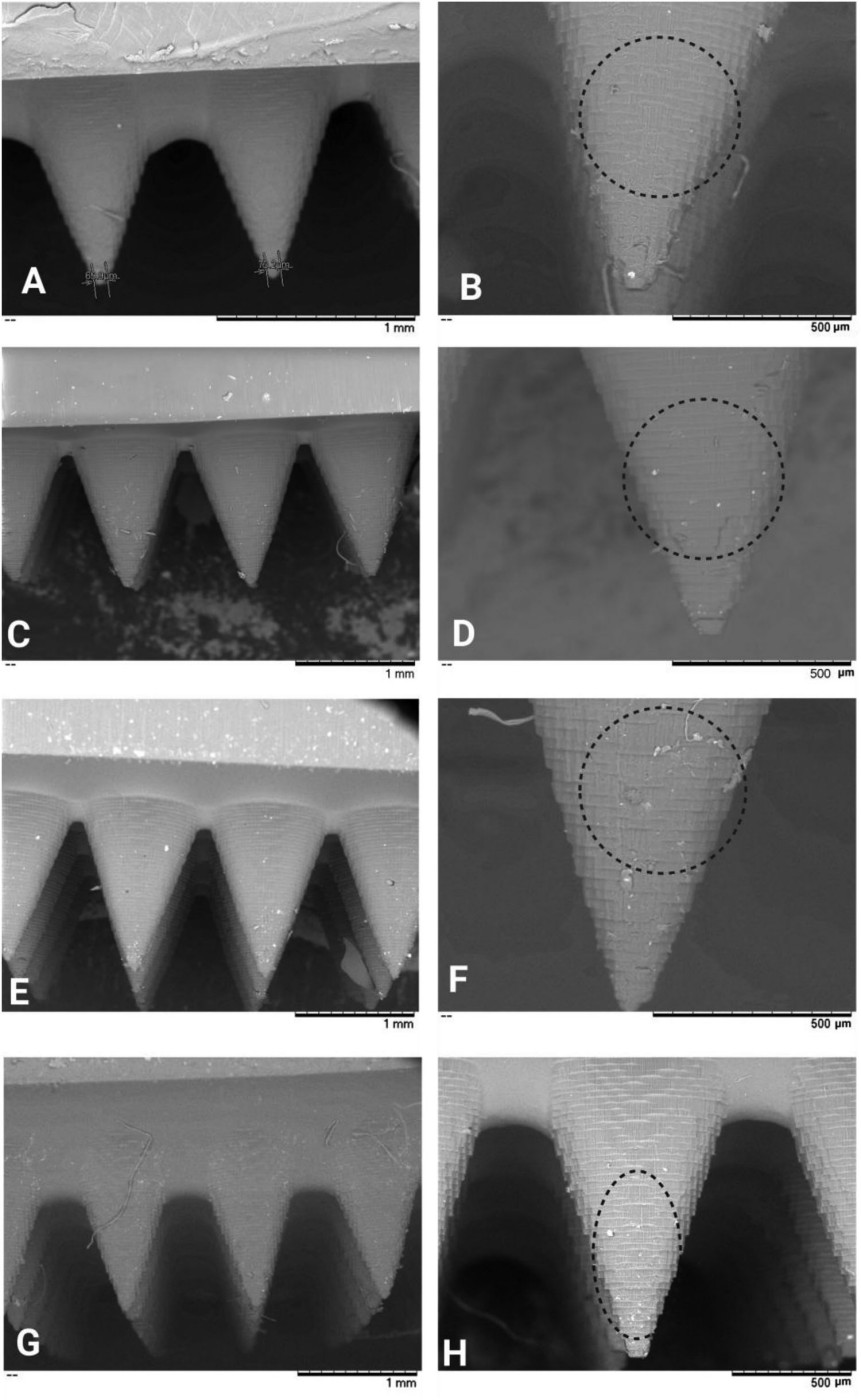
SEM MNs morphology and tip details. (A and B) placebo; (C and D) 5‐FU; (E and F) CUR; (G and H) CUR+5‐FU. Left images show the overall needle geometry; right images show the tip morphology. The layer‐by‐layer topography from DLP printing is visible, and the tips are uniformly sharp. Occasional surface particulates on drug‐loaded MNs were consistent with API entrainment.

The SEM images (Figure 6) reveal that the printed MNs possessed the expected layer‐by‐layer texture characteristics for DLP fabrication (“staircase effect”) with micron‐scale surface roughness. This moderate roughness, though inherent to the printing process [43], is beneficial: it increases the skin contact area, enhances adhesion, and facilitates fluid uptake during dissolution, potentially improving drug release kinetics [44]. The tips of all MNs were uniformly sharp, which is an essential feature for effective penetration of the stratum corneum. Drug‐loaded formulations (5‐FU, CUR, and CUR + 5‐FU) retained their overall shape but exhibited minor surface particulates, consistent with partial localization of the API near the matrix surface. Placebo MNs appeared smoother in addition to the layered texture. The patch mass remained consistent across formulations, with low standard deviations (≤ 1.5 mg). Drug content analysis confirmed values close to the theoretical loadings, demonstrating excellent dosing reproducibility and manufacturing uniformity. Collectively, these results verify that DLP fabrication enables high‐resolution replication of complex MN geometries and that the PEGDA/VP photo resin maintains structural precision, even with drug incorporation. The resulting arrays combine sharp, well‐defined tips and controlled roughness, attributes critical for consistent skin penetration and predictable release behavior, making this dual‐drug MN system a robust platform for localized transdermal chemotherapy.

### 2.8 Insertion Performance on a Parafilm Skin Model

**FIGURE 7:**
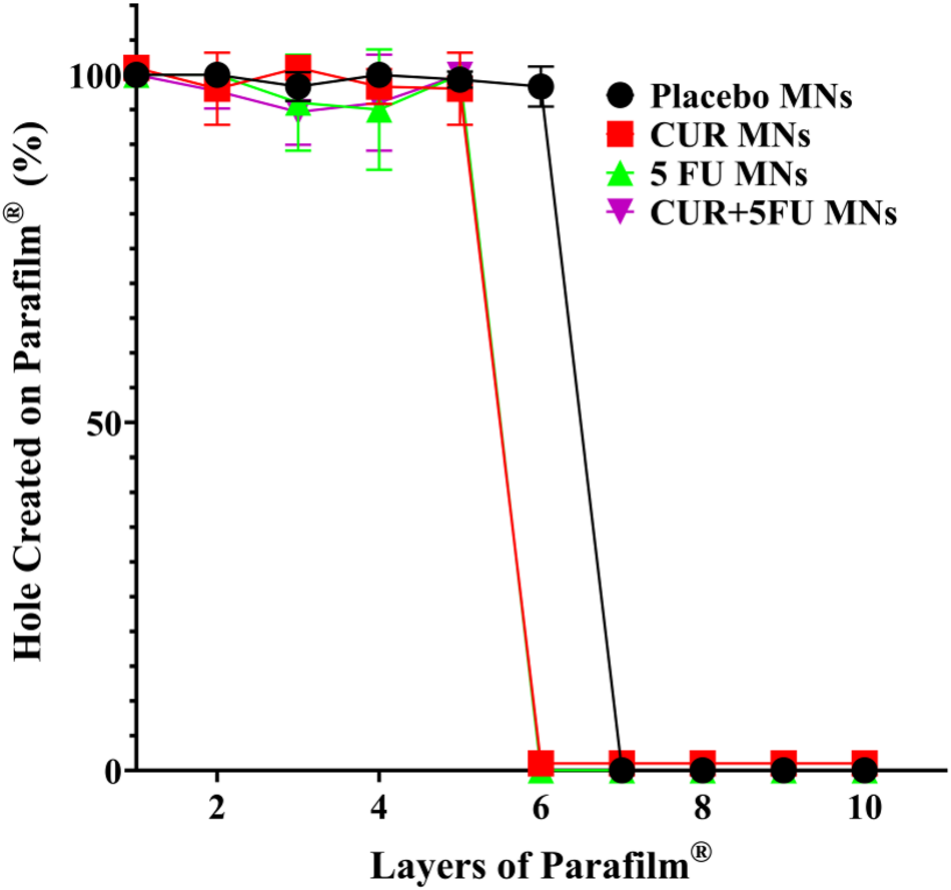
Insertion efficiency of the MN patches measured by texture analysis. Percentage of holes created in successive Parafilm layers by MNs insertion under a standard force; values are mean ± SD (*n* ≥ 5) for placebo, 5‐FU, CUR, and CUR+5‐FU formulations.

The ability of MN arrays to create reliable microchannels in the skin is a critical determinant of their therapeutic efficacy. To assess mechanical robustness and insertion efficiency, a 10‐layer Parafilm stack (∼1 mm total thickness) was used to simulate the stratum corneum and upper dermis. Hole formation in each layer was quantified as the percentage of needles creating pores, and needle height was measured before and after insertion to evaluate deformation. Each MN patch was pressed into the stack using a texture analyzer under a standardized axial force. Placebo MN arrays achieved 100 % penetration through six Parafilm layers, while 5‐FU‐, CUR‐, and dual‐loaded MNs achieved 100 % penetration through five layers (Figure 7). In all cases, microchannel formation exceeded 90 % for the first five layers, confirming that both the drug‐free and drug‐loaded arrays robustly perforated the simulated stratum corneum and viable epidermis. This performance compares favorably with that of typical polymeric MNs reported in the literature. For example, the author observed ∼60 % insertion of hydrogel‐forming MNs through Parafilm [45]. More recently, researchers reported that a polyvinylpyrrolidone MN design was inserted completely through the fourth Parafilm layer (∼440 µm, ≈90 % of needle length), whereas earlier arrays often penetrated only 2–3 layers [45]. Thus, the PEGDA/VP‐based MNs in this study provided deeper and more consistent insertion than many existing systems, despite co‐loading with hydrophilic (5‐FU) and hydrophobic (CUR) drugs. Optical microscopy showed that the needle height decreased by ≤1% after instrumented insertion, indicating that the functional mechanical performance of the arrays was sufficient to withstand the applied force without buckling or fracture. Manual “thumb press” testing (≈20 N force for 30 s) produced similar results: all formulations penetrated through at least six layers, and average needle height decreased by only ∼0.5 %. These findings suggest that end‐users could easily apply the patches without special devices and that co‐loading the two drugs did not compromise insertion efficiency.

The enhanced insertion depth observed here can be attributed to several design features: (1) Higher needle length (1.5 mm) than many dissolving MN designs; (2) Optimized PEGDA/VP resin composition that imparts mechanical strength and shape fidelity; and (3) Sharp tips and controlled surface roughness resulting from pixel‐aware DLP printing (see Morphology section). While many studies demonstrate effective delivery using MNs that penetrate only a few Parafilm layers, deeper penetration increases the likelihood of crossing the stratum corneum and reaching intradermal tumor regions. Moreover, compared with the work of [45], who required multiple experimental setups to reach ∼90 % of their MN length, the arrays reported here achieved full penetration across a larger number of layers using a single standardized test, demonstrating superior mechanical performance. Taken together, these results establish that the dual‐drug DLP‐printed MNs possess the necessary mechanical robustness and insertion capability for reliable transdermal penetration, surpassing those of many previously reported polymeric systems. The co‐loaded array‐maintained insertion efficiency was comparable to that of placebo MNs, underscoring that incorporation of both 5‐FU and CUR did not compromise mechanical properties. This combination of high penetration depth and user‐friendly application reinforces the translational potential of the PEGDA/VP MN platform for localized chemotherapeutic delivery.

### 2.9 Ex Vivo Penetration into Soft and Skin‐Like Tissues

**FIGURE 8:**
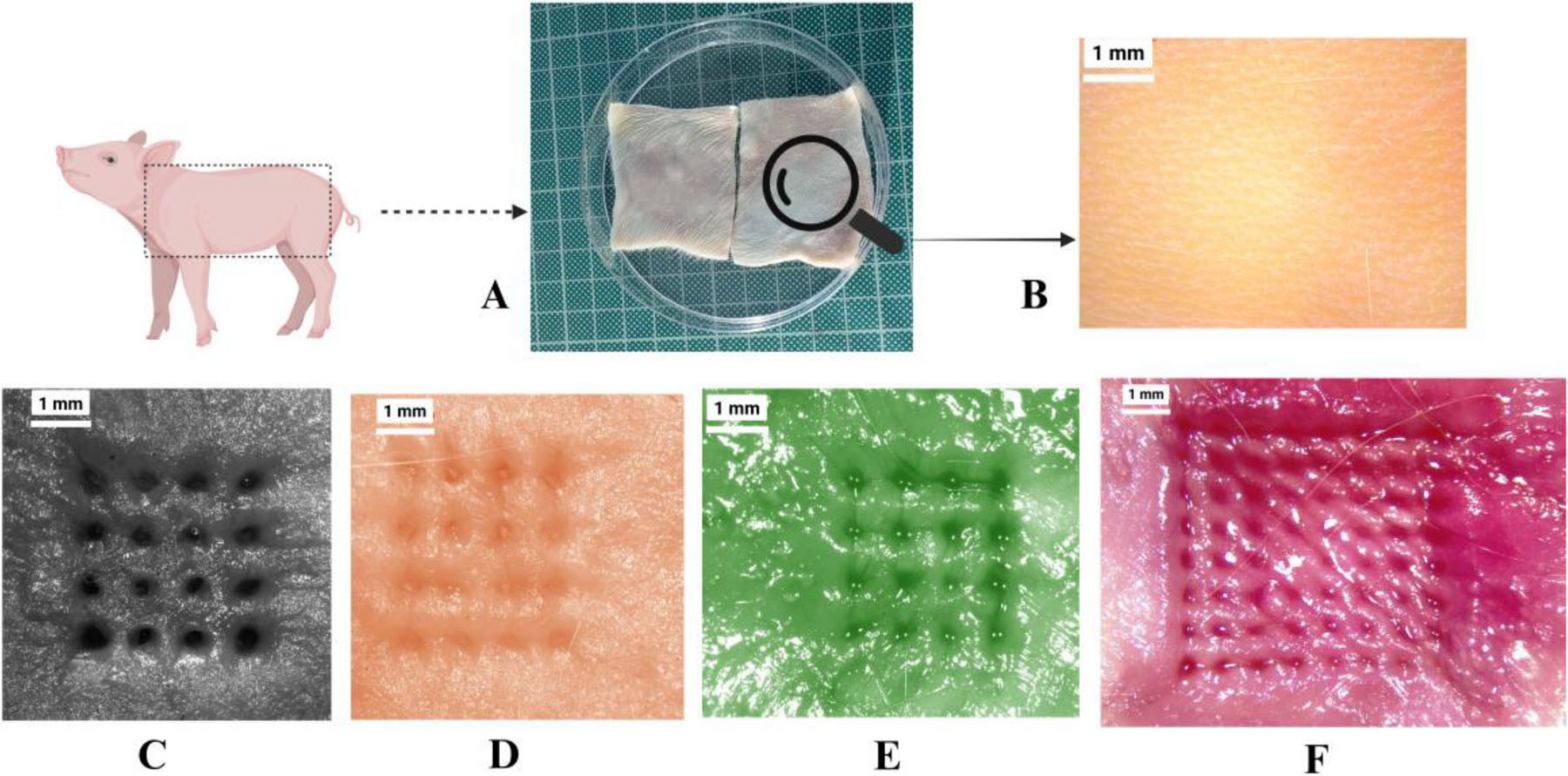
Ex vivo MNs insertion in porcine skin (stillborn piglet) with optical micrographs of the tissue before and after MNs application. Panels (A) and (B) show untreated pork skin and skin surface just before insertion, respectively. Panels (C–F) show the resulting micropores (holes) formed by MN patches: (C) placebo MNs, (D) CUR MNs, (E) 5‐FU MNs, and (F) MNs loaded with both CUR and 5‐FU.

**FIGURE 9:**
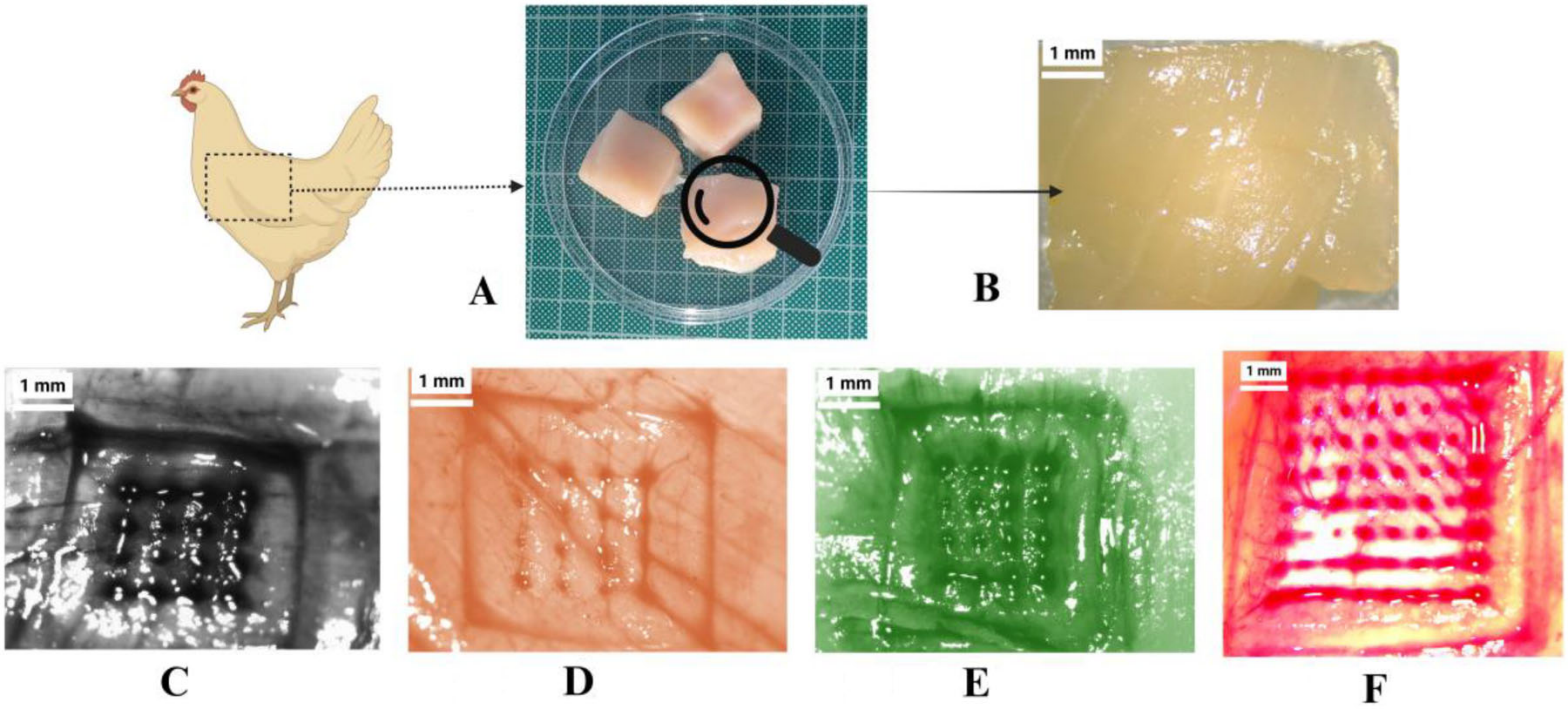
Ex vivo MNs insertion in chicken breast tissue with optical micrographs taken before and after MN application. Panels (A and B) show untreated chicken tissue and the tissue surface before insertion, respectively. Panels (C–F) show the micropores created by (c) placebo MNs, (d) CUR MNs, (e) 5‐FU MNs, and (f) MNs co‐loaded with CUR and 5‐FU^1^.

To assess real‐tissue performance, MN arrays were applied ex vivo to both chicken breast muscle and neonatal porcine skin. These substrates model soft muscles and fibrous skin [46]. The MN patches were manually pressed into the tissues, and puncture formation, needle integrity, and qualitative penetration depth were examined using stereomicroscopy (Figures 8 and 9). In the compliant chicken breast model, both placebo‐ and drug‐loaded MNs produced well‐defined puncture marks arranged according to the array geometry. The needles penetrated cleanly and were removed intact with no evidence of shank buckling or tip breakage. Although quantitative insertion depth was not measured, the visible microchannels and lack of residual drug on the tissue surface suggest insertion approaching the full 1.5 mm needle length (∼0.5 mm depth in the soft muscle). Comparable studies using conical polymer MNs in chicken breast required ∼813 mN maximum force for full penetration [46], whereas the prepared patches achieved complete insertion via simple manual pressure, underscoring the mechanical robustness of the PEGDA/VP resin and the sharp needle geometry. Similarly, neonatal porcine skin, which more closely mimics the layered structure and mechanical stiffness of human skin, also showed consistent entry of each MN. Distinct micropunctures were observed, and needles emerged without deformation. The tougher substrate is likely to have a reduced penetration depth relative to chicken muscle, but the formation of discrete pores confirms that the arrays breached the stratum corneum and entered the viable dermis. Literature reports on polymeric MNs indicate that insertion forces of 4.4 N per array (∼330 µm penetration) up to 16.4 N per array (520 µm penetration) are required to pierce neonatal porcine skin [47].

Furthermore, increasing the needle spacing from 30 to 150 µm reduced the insertion force from 0.030 N to 0.028 N per needle. In the current study, MNs achieved full penetration under a manual force (∼20 N applied to the entire patch) despite containing two APIs, highlighting that the optimized PEGDA/VP formulation and 1.5 mm needle length provide sufficient functional mechanical performance and sharpness for insertion without requiring high insertion forces. These ex vivo results reinforce the importance of needle geometry and material selection. Sharp tips, appropriate aspect ratio, and a mechanically robust PEGDA/VP network ensured that neither 5‐FU nor CUR compromised insertion efficiency.

### 2.10 Swelling and Degradation Behavior of MN Arrays

**FIGURE 10:**
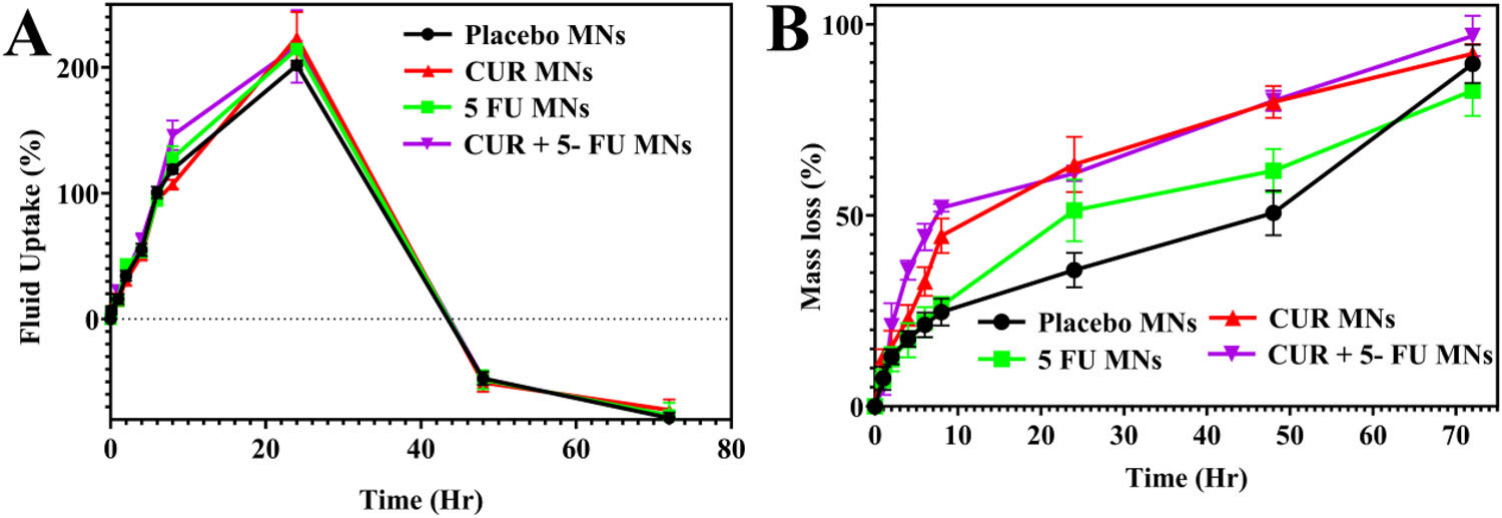
(A) Time‐dependent swelling of placebo, 5‐FU, CUR, and CUR+5‐FU MNs in PBS. Curves represent percentage weight gain relative to the initial mass (mean ± SD, *n* ≥ 3). (B) Degradation (mass loss) profiles of the same formulations, plotted as percentage of original mass remaining over time (mean ± SD, *n* ≥ 3).

The swelling and degradation profiles of the PEGDA/VP MN patches (placebo, 5‐FU, CUR, and CUR + 5‐FU formulations) revealed rapid and distinct two‐phase behavior upon immersion in phosphate‐buffered saline (PBS). A pronounced initial swelling phase occurred within the first six hours, reflecting the high hydrophilicity of the VP component, which drives water uptake through hydrogen bonding with the polymer network already investigated study [26]. This observation aligns with earlier findings by [14], who reported that VP‐rich PEGDA/VP systems reached maximal expansion by approximately six hours, and with [12], who observed similarly rapid hydration in DLP‐printed PEGDA/VP MNs. The intense early swelling indicates that the network readily absorbs fluid from its environment, enhancing the diffusion of encapsulated drugs. After this initial phase, the MNs exhibited a slow reduction in swollen weight, noticeable after 24 h, consistent with the saturation of the medium and the onset of hydrolytic cleavage within the crosslinked matrix (Figure 10A). As the networks approached equilibrium hydration, minor erosion occurred, which was likely due to polymer chain scission and fragment detachment. Degradation assays (Figure 10B) confirmed progressive mass loss over 72 h, with placebo MNs retaining approximately 15% of their initial mass, whereas 5‐FU, CUR, and CUR + 5‐FU formulations lost ∼80%, ∼90%, and nearly 100% of their mass, respectively. This gradual erosion pattern is typical of PEGDA/VP‐based materials, where water uptake precedes the solubilization of the polymer chains. The correlation between swelling kinetics and mass loss highlights a dual‐release mechanism: a rapid initial phase driven by water absorption and polymer relaxation, followed by a slower, sustained phase governed by matrix degradation. Collectively, these findings demonstrate that the developed PEGDA/VP MNs combine rapid hydration for prompt drug mobilization with controlled structural degradation, thereby supporting their potential for sustained transdermal drug delivery.

### 2.11 % Drug Content and In Vitro Release

In this study, the single‐drug MN formulations, the PEGDA‐VP resin contained 1.5 wt% 5‐FU or 1.0 wt% CUR, yielding a 100 mg patch incorporating 1.5 mg of 5‐FU or 1.0 mg of CUR within the 6 × 6 array design. In the dual‐drug configuration, the resin was loaded with 1.0 wt% 5‐FU and 0.5 wt% CUR, producing a 10 × 10 patch weighing approximately 250 mg and containing 2.5 mg of 5‐FU and 1.25 mg of CUR, consistent with the increased drug‐dose capacity afforded by the larger patch geometry. These concentrations were chosen by considering the marketed and experimental formulations mentioned in Table 3. Commercial 5‐FU products include 0.5 %, 1 %, 2 %, and 5 % creams or solutions. For actinic keratosis, 0.5 % cream (e.g., Carac) is typically applied once daily to cover the lesion area for up to 4 weeks [48]. Similarly, higher‐strength formulations such as 1 %, 2 %, or 5 % creams/solutions (e.g., Efudex/Efudix) are applied twice daily for 2–4 weeks, continuing until the inflammatory response reaches the erosion stage. These topical treatments rely on prolonged surface contact; removal or washing can reduce the effective dose. Reported topical CUR formulations, such as 0.5 % microemulgel and 1 % alcoholic gel, have been used experimentally for conditions like psoriasis [49], yet they suffer from limited dermal penetration and susceptibility to light‐induced degradation. To provide localized drug exposure and reduce reliance on surface residence, MNs were designed to bypass the stratum corneum and deliver APIs directly into the viable epidermis and dermis. The selected concentrations in MNs therefore reflect lower loadings than marketed 5 % 5‐FU creams but remain within the range of concentrations shown to be clinically relevant. For dual‐drug patches, a resin combination of 1.0 wt % 5‐FU and 0.5 wt % CUR was used, enabling concurrent delivery of a cytotoxic agent and an anti‐inflammatory/antioxidant agent. Intradermal deposition reduces the need for repeated dosing and minimizes the risk of drug wipe‐off, while CUR susceptibility to photodegradation is mitigated by its encapsulation within the polymer matrix. The formulation strategy thus aligns with established topical concentrations while taking advantage of MN‐mediated intradermal delivery.

**TABLE 3:** Comparative overview of MN and topical formulations of 5 FU and CUR, including dose strengths, patch/cream geometry, delivery modality, and reported outcomes.

Delivery	Strength (mg per patch or % w/w)	Patch geometry/array	Delivered dose or % release	Reference
Bilayer MN co‐loaded with triamcinolone and 5‐FU	Drug loading equivalent to 58 µg 5‐FU and 52 µg triamcinolone per patch.	Conical MN array; two‐layer structure	Release profile: ∼50 % of both drugs released within 12 h; significantly prolonged drug retention.	[50]
MN patch	Dose in dissolving MN patch reported as (0.375 mg in the MN portion and 14.625 mg in the base)	Not reported	Clinical outcomes reported; delivered mass, % release not reported	[51]
Topical cream	0.5% w/w FU, applied once daily as a thin film (exact mass applied is patient‐dependent)	Not applicable	Systemic exposure context reported in label; local delivered dose not reported	[52]
Topical cream	5% w/w FU cream (e.g., Efudex) used twice daily up to 28 days in a PK study	Not applicable	Local delivered dose not reported	[52]
MN patch	CUR content in MN array: 10.9 ± 1.1 µg per array	11 × 11 conical MNs; height ≈900 µm; base diameter ≈300 µm	MNs reported to penetrate neonatal porcine skin (∼500 µm) and dissolve within ∼60 min; deeper drug diffusion compared with the topical comparator (quantitative applied dose not reported)	[53]
Topical comparator	Topical CUR nanosuspension (exact applied mass or percentage not reported in abstract)	Not applicable	Drug distribution reported as lower and less deep than MN delivery (quantitative applied dose not reported)	[53]

Additionally, the drug load delivered by an MN patch can be modulated by altering its geometry, a capability particularly useful for achieving therapeutic concentrations in cancer treatment. The increase in patch area and mass allows greater resin volume and thus a higher total drug mass without altering the polymer‐to‐drug ratio or compromising print fidelity. By varying needle count, height, base diameter, and patch footprint in the CAD model, the DLP printer can generate personalized geometries tailored to tumor size or skin area, enabling dose escalation or reduction according to the required therapeutic concentration. This geometric flexibility, combined with the direct intradermal delivery of MNs and the high precision of DLP printing, offers a promising pathway for customizing transdermal chemotherapy patches beyond the fixed doses typical of standard topical formulations.

**FIGURE 11:**
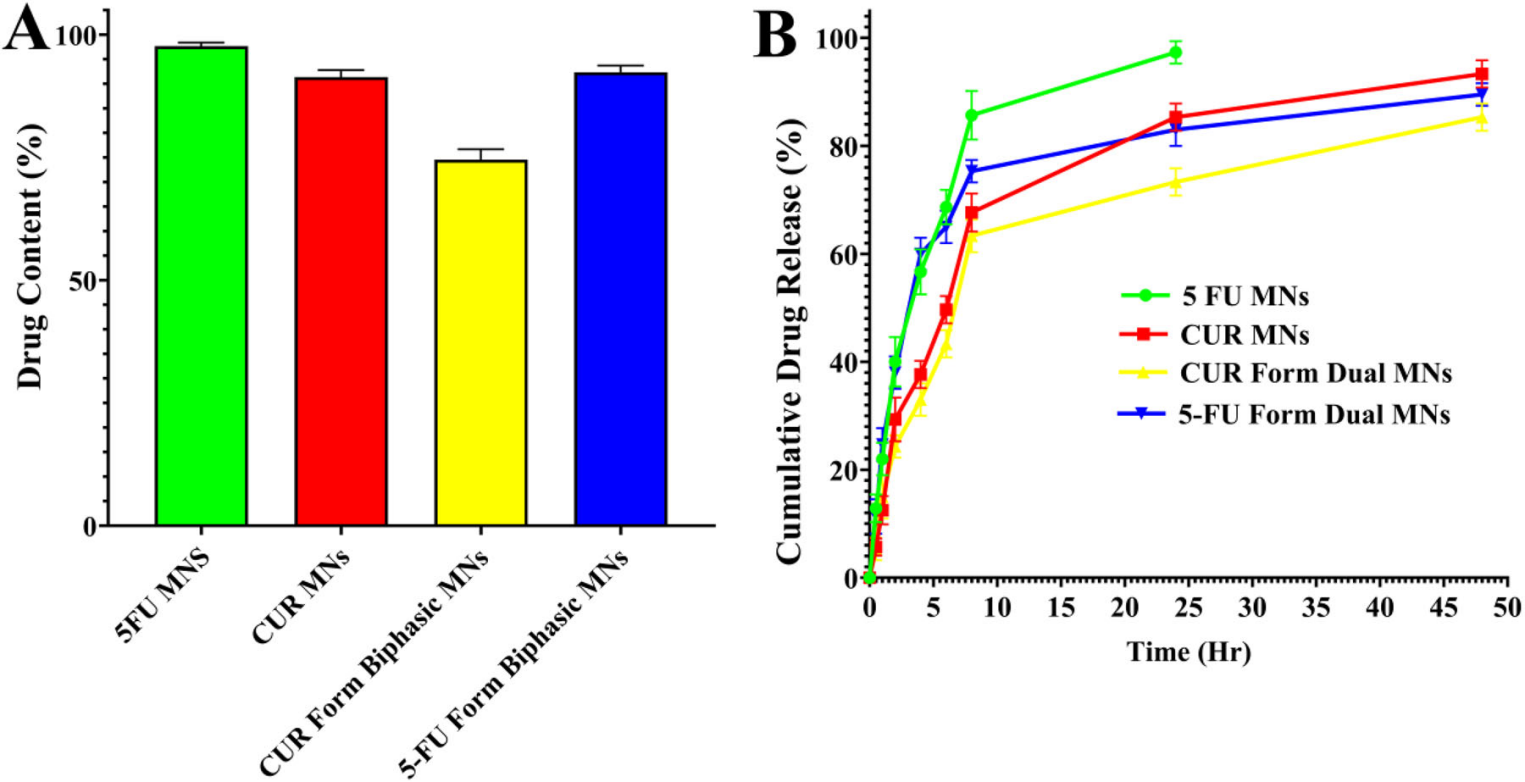
(A) Percentage Analysis of Drug Content in MNs for 5‐FU, CUR, and CUR+5‐FU (mean ± SD *n* ≥3 for all formulations). (B) Drug Release Analysis for 5‐FU, CUR, and CUR+5‐FU MNs by HPLC.

Drug‐content assays confirmed high entrapment of both actives in the PEGDA/VP MNs using high‐performance liquid chromatography (HPLC). During the HPLC method development, the calibration curve was successfully established, and the corresponding LOD and LOQ values were determined. Samples analyzed by HPLC revealed that the CUR‐loaded MNs retained 92 ± 2 % of the theoretical dose, while 5‐FU‐loaded MNs achieved 97 ± 1.5 % recovery; in the combination patches, CUR entrapment was slightly lower (75.25 ± 2 %), but 5‐FU remained high (92.21 ± 1.0 %) (Figure 11A).

Release studies under sink conditions at 32 °C bottle dissolution method showed that from an individual drug containing a patch 6 × 6 array, CUR exhibited a biphasic profile: a burst within the first 4 h, driven by rapid swelling of the VP‐rich matrix, followed by sustained diffusion, delivering 96 ± 0.6 % over 48 h. It should be noted that CUR and 5‐FU are incorporated within the same single‐matrix PEGDA/VP photopolymer network rather than in separate layers; the term “biphasic” therefore refers to the release behavior arising from hydration, drug diffusion, and matrix erosion/degradation, and not from layer‐by‐layer drug compartmentalization. This initial surge arises from the hydrophilic VP fraction, which rapidly imbibes water and opens diffusion channels. In contrast, highly soluble 5‐FU displayed a smoother, controlled release, with 99% ± 0.5 % released within 24 h. In the dual‐drug formulation, the cumulative release of each drug after 48 h was slightly reduced (CUR ≈85 %, 5‐FU ≈90 %), reflecting a combination of two API loading in one MN patch, and slower water uptake (Figure 11B). As the dual‐drug MN patch was fabricated in a larger 10 × 10 array, its increased structural volume and polymeric mass may have contributed to a slower release profile than that of the smaller single‐API patches. After 48 h, the release profile approached a steady‐state phase, indicating a reduction in the rate of drug diffusion from the polymeric matrix. These profiles reveal an interplay between swelling kinetics and drug solubility; hydrophobic CUR responds to rapid matrix hydration with a pronounced early release, whereas hydrophilic 5‐FU diffuses steadily through the hydrated network. When fitted to kinetic models, 5‐FU release showed high agreement with first‐order, Higuchi and Korsmeyer–Peppas models (*R* ^2^ ≥ 0.98), indicating diffusion‐driven release coupled with concentration‐dependent kinetics; the Peppas exponent (0.5 ≤ *n* < 1) suggested primarily Fiskian diffusion. CUR release conformed best to Higuchi and Korsmeyer–Peppas models, consistent with diffusion‐controlled transport. The co‐loaded patches exhibited kinetic parameters comparable to those of single‐drug systems, confirming that co‐loading did not alter the underlying diffusion mechanism. A comparison with similar MN systems highlights the advantages of the proposed approach. Bilayer dissolving MNs co‐loaded with 5‐FU and triamcinolone achieved only ∼50 % permeation of each drug through scar tissue after 12 h, and when 5‐FU was encapsulated in the slow‐swelling tail layer, the cumulative permeation at 12 h was just 8.34 ± 4.51 % [50]. In contrast, the PEGDA/VP MNs reported here released 99 % of 5‐FU within 24 h by using the vial dissolution method from 6 × 6 array, demonstrating more complete liberation from a single‐layer matrix. Similarly, hyaluronic acid MNs delivering CUR nanocrystals achieved greater penetration than gel formulations but still depended on particle size; only the smallest CUR‐NCs showed a two‐fold increase in cumulative release after 48 h compared with gel [54]. In the current system, despite delivering free CUR rather than pre‐formed nanocrystals, 96% of the drug was released within 48 h, indicating comparable or superior total release via dissolution without requiring nanocrystal fabrication. Collectively, these findings confirm that the PEGDA/VP MNs afford high drug entrapment and biphasic release, with diffusion‐controlled kinetics and enhanced performance relative to other 5‐FU and CUR MN platforms. However, it should be recognized that dissolution testing measures the upper limit of drug availability; transdermal diffusion in skin will likely yield lower cumulative permeation. The results here therefore provide a mechanistic understanding of drug liberation from the matrix and emphasize the need for subsequent ex vivo and in vivo studies to establish therapeutic relevance.

The therapeutic significance of the present system therefore lies primarily in its delivery performance rather than in claiming a new drug combination. Previous work has already shown that CUR and 5‐FU can be applied using MN‐mediated strategies [22]. The added value here is that both agents are incorporated within a single dissolving DLP‐printed PEGDA/VP matrix that supports localized intradermal deposition, minimizes dependence on surface residence, and allows the total drug payload to be adjusted by modifying the CAD‐defined array geometry. This approach may reduce limitations associated with surface‐coated solid MNs, including coating non‐uniformity, incomplete transfer, and potential drug loss before or during insertion. Nevertheless, the present data should be interpreted as formulation and performance evidence. The study does not demonstrate therapeutic synergy or superiority in a skin‐cancer model, and future work should directly assess cytotoxicity, combination index behavior, skin permeation, tumor penetration, and in vivo antitumor efficacy.

### 2.12 Stability Testing of Resin and MNs

The pragmatic stability study compared the behavior of the PEGDA‐VP resin and the printed MN arrays under different storage conditions (low temperature vs. room temperature vs. stress, with and without light protection). The resin samples stored under low temperature (4 °C) in light‐protected containers remained clear and could be reused for printing for at least 60 days; although the PEGDA component gelled during freezing, it returned to a free‐flowing liquid after thawing and performed normally in test prints. These observations are in line with recommendations in the literature that photopolymerizable PEGDA formulations should be stored at low temperature and protected from light to prevent premature cross‐linking [55, 56]. For example, PEGDA hydrogels prepared with LAP are stored at 4 °C in the dark, and supplier guidance for PEGDA recommends storage at 2–8 °C and protection from light. In contrast, unprotected resin stored at ambient laboratory temperature (20–25 °C) began to show evidence of spontaneous polymerization within 15 days and fully solidified by 60 days, reflecting the propensity of acrylate‐based resins to undergo uncontrolled curing when exposed to room‐light and heat. This behavior was also observed for 5‐FU‐ and CUR‐loaded resins, emphasizing that light‐protected, low‐temperature storage is critical for maintaining printability.

Printed MN arrays exhibited good stability when stored at room temperature in the absence of light: placebo and API‐loaded MNs maintained their physical dimensions, mechanical strength and drug content for the full 60‐day period. This finding is consistent with reports that dissolving MN patches stored at 4 °C or 25 °C show no detectable changes in appearance or payload over several months [57]. When MNs were stored at room temperature in open containers, the percentage assay of CUR declined, and the patches became brittle and discolored, whereas 5‐FU content remained largely intact despite some physical roughening of the array. The greater degradation of CUR is attributed to its well‐known photolability; curcuminoids are stable to heat but undergo rapid degradation and decolorization when exposed to sunlight [58]. Temperature‐stress tests at 37 °C confirmed this trend: light‐protected MNs showed no significant changes, but exposed arrays exhibited cracking and reduced structural integrity, underscoring the importance of shielding MNs from both light and elevated temperature during storage. Overall, the results demonstrate that appropriate storage‐freezing or refrigeration for the liquid resin and light‐protected ambient conditions for the solid MN patches are essential to preserve printability, mechanical strength and drug potency.

## 3 Conclusion

This study demonstrated that DLP 3D printing can be used to fabricate high‐fidelity dissolving MN arrays for the co‐delivery of CUR and 5‐FU from a single PEGDA/VP photocurable matrix. The work is distinct from prior CUR/5‐FU MN reports, particularly inkjet/coating‐based systems, because both drugs were incorporated directly into the printable resin before fabrication rather than deposited onto preformed solid MNs. This distinction is important because direct DLP printing integrates formulation composition, photopolymerization behavior, CAD‐defined geometry, dose capacity, insertion performance, matrix hydration/degradation, and drug release into one manufacturing workflow. By optimizing a PEGDA/VP (40:60 w/w) photo resin containing 0.4% LAP, printable inks with low viscosity, rapid curing, and strong adherence to CAD specifications were obtained. The resulting MN arrays maintained sharp tip geometry and tolerated incorporation of both drugs without major deformation. Fine‐tuning of exposure parameters compensated for CUR light absorption, ensuring consistent polymerization across placebo, single‐drug, and dual‐drug formulations. Comprehensive characterization confirmed that the material design and process control translated into robust performance. Placebo‐ and drug‐loaded MNs showed near‐quantitative insertion in Parafilm and ex vivo skin models with negligible post‐insertion deformation, indicating that co‐loading did not compromise functional insertion performance; however, direct mechanical characterization of the cured polymer network, such as compression testing, tensile testing, dynamic mechanical analysis, or oscillatory rheology of the cured hydrogel, should be addressed in future work. Swelling and degradation studies revealed a two‐stage behavior: rapid hydration driven by the VP component followed by gradual mass loss through matrix erosion. These dynamics produced biphasic release profiles, with high entrapment efficiencies and diffusion‐dominated release kinetics supporting predictable liberation of both actives. Overall, the novelty of this work lies in the formulation‐process‐performance integration of a directly DLP‐printed dual‐drug dissolving MN platform, rather than in the first use of CUR and 5‐FU together. The arrays offer precise geometry, localized delivery, tunable payload through CAD‐defined patch design, and reduced reliance on surface coating or prolonged topical residence. A further limitation is that the study did not include dedicated biological safety or therapeutic efficacy testing; future work should assess skin irritation, cytocompatibility, local inflammatory response, histological tissue response, post‐MN skin recovery, skin‐cancer‐specific cytotoxicity, combination effects, and in vivo antitumor performance before translational development.

Similarly, the realistic views on study, DLP printing offers inherent scalability because each exposure cures an entire layer, allowing multiple MN patches to be manufactured in parallel. However, transitioning from laboratory to industrial scale requires strict control over printing parameters, such as optical dose per layer, feedstock preparation, and resin viscosity, to ensure consistent geometry and mechanical strength across batches. On a commercial scale, a robust quality management system is essential: printers must be validated for accuracy, cleanliness and reliability, and processes should adhere to current Good Manufacturing Practice. Dissolving MNs fall under the category of combination products, meaning regulatory review must address both the device and the incorporated drugs. Existing guidance for microneedling devices emphasizes product classification, sterility assurance, and the need to evaluate any therapeutic claims separately from cosmetic use. Because AM frameworks are still evolving, it is prudent to engage early with regulatory authorities, adopt a quality‐by‐design approach, and establish thorough documentation of critical material attributes and process controls. Addressing these factors will help transform promising proof‐of‐concept MN into scalable, patient‐ready products that meet regulatory expectations for safety, efficacy and quality.

## 4 Experimental Section/Methods

### 4.1 Materials

CUR and 5‐FU (≥99%) were sourced from TCI (Japan). VP (≥98%), PEGDA with a molecular weight of 700 g/mol, and LAP (≥95%) were obtained from Sigma Aldrich, part of Merck KGaA (Darmstadt, Germany). Additional chemicals, methanol (MeOH, ≥99.9%), acetonitrile (ACN, ≥99.9%), acetic acid (≥99 %), and trifluoroacetic acid (TFA; ≥99.0 %) were also included. PBS tablets at pH 7.4 were included as well. Nile Red (≥97%) was obtained from Merck KGaA (Darmstadt, Germany). The water used in the experiments was deionized, distilled, and subsequently filtered through a Millipore Q purification system.

### 4.2 Preparation of PEGDA/VP Resins Loaded with API

Photocurable resins were formulated by incorporating CUR, 5‐FU, and the photoinitiator LAP into the PEGDA and VP matrices. Precisely weighed amounts of CUR, 5‐FU, and LAP were dispersed in PEGDA and triturated to obtain homogeneous suspensions. VP was then gradually added under continuous vortex mixing to yield a clear, uniform solution. Single‐drug formulations (CUR‐only and 5‐FU‐only) and dual‐drug resins with a combination of (CUR + 5‐FU) were prepared following the same procedure, maintaining a PEGDA: VP mass ratio of 40:60 (w/w), as established in prior optimization studies [26]. The LAP concentration was fixed at 0.4 wt% relative to the total resin mass. All resin preparations were conducted under low‐light conditions to prevent premature photopolymerization. The resulting mixtures were stored in amber containers at room temperature and used within 48 h of preparation. Rheological analysis was performed to evaluate printability, including steady‐shear viscosity measurements and shear‐rate flow profiling (10‐100 s^−^^1^). FTIR spectroscopy (ATR mode, 4000–500 cm^−^^1^, 4 cm^−^^1^ resolution) was employed to verify photopolymerization by comparing the spectra of uncured and light‐cured samples.

### 4.3 Flow Behavior and Rheological Analysis of Resins

Evaluating the viscosity of the resin is crucial for successful printing, as higher viscosity can lead to printing defects. The resin viscosity was measured using a Rheometer (Thermo Fisher Scientific, Loughborough, UK). The shear rate test was conducted using a parallel plate geometry with a diameter of 35 mm, over a time range of 0.0 to 100 s^−^^1^, at a controlled room temperature of 20 °C as specified in the protocol [31]. Each sample from the placebo and API loaded (5‐FU and CUR) resin was evaluated in triplicate to ensure accuracy and reliability. Viscosity measurements were taken as the shear rate was applied, with viscosity calculated once the samples reached a steady‐state, or plateau, phase.

### 4.4 DLP Fabrication of Single and Dual Drug MN Arrays

**FIGURE 12:**
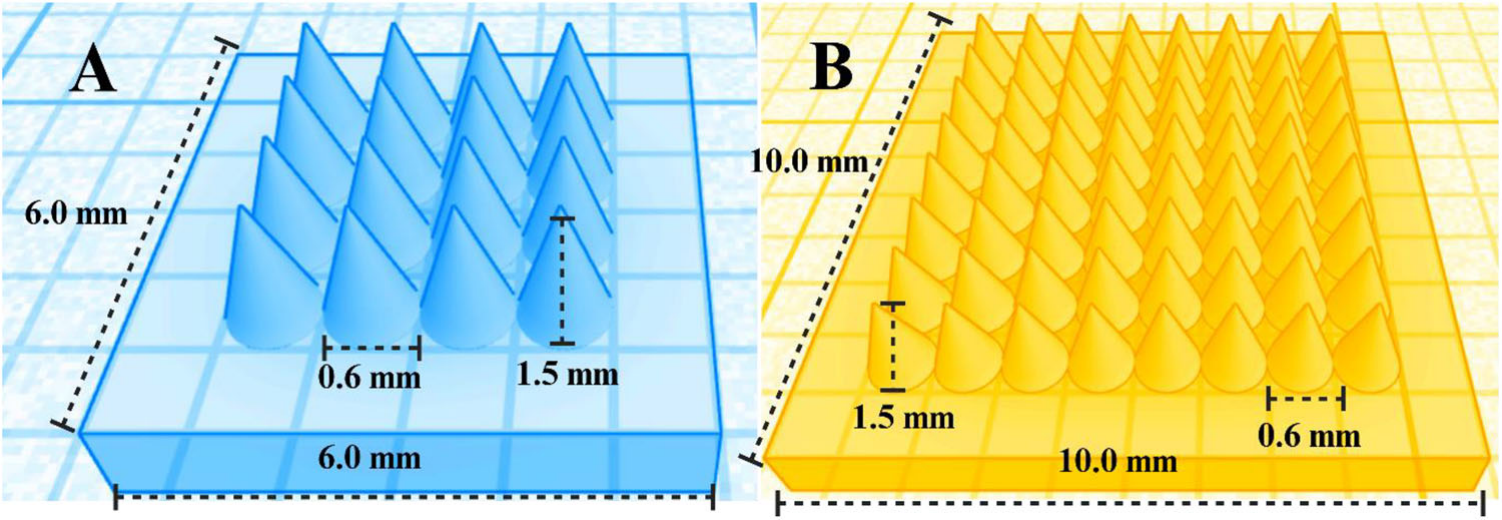
Computer‐aided design of MNs patch geometries for DLP printing, (A) 6 × 6 × 1.5 mm and (B) 10 × 10 × 1.5 mm (width × length × thickness); models exported as stereolithography (STL) files for slicing and build preparation.

MN array geometries were designed in Tinkercad (Autodesk, San Francisco, CA, USA). The different patch variants were generated from two of them in Figure 12: (A) placebo and single‐API formulations, and (B) a combination patch co‐loading with CUR and 5‐FU in which both drugs are homogeneously dispersed in a single matrix, in a DLP 3D printer (Asiga Max X27, Asiga, Sydney, NSW, Australia). CAD models were exported as stereolithography (STL) files, imported into Asiga Composer for slicing software (version 1.2.11) and built preparation, and fabricated on a DLP 3D printer using the optimized resin composition described in the results section. Additionally, to find out the optimum concentration of the drug, the trials were conducted using varying API concentrations tailored to the MN designs. The printing parameters (layer thickness, exposure time, light intensity, and build orientation) were reported. As they play a fundamental role in governing photopolymerization behaviors and dimensional accuracy of the MN structures. Variables such as layer thickness and exposure time influence vertical resolution and curing depth, while light intensity and build orientation affect tip sharpness and array uniformity. Understanding these factors is essential for identifying conditions that yield reliable and mechanically robust MNs. Their inclusion also enhances methodological transparency and facilitates reproducibility of the fabrication process. Following fabrication, the printed parts were rinsed to remove unpolymerized resin. MNs were inspected for gross artefacts (e.g. incomplete tips, warping, and residual resin) before downstream characterization.

### 4.5 Spectroscopic Confirmation of Drug Polymer Network

ATR‐FTIR spectroscopy was used to characterize API‐polymer interactions and to verify photopolymerization of the PEGDA/VP system. A physical mixture was prepared by vortex‐mixing the relevant components to homogeneity, and spectra were acquired for the neat APIs, individual polymers, uncured resin, and UV‐cured MNs. ATR‐FTIR spectra (Thermo Scientific Nicolet iS50, Paisley, United Kingdom) were recorded over 4000–400 cm^−^^1^ at 4 cm^−^^1^ resolution with background subtraction; each sample was measured in triplicate to ensure repeatability. Polymerization was assessed by monitoring the depletion of acrylate vinyl bands in cured matrices together with the relative growth‐intensification of backbone carbonyl absorptions characteristic of the cross‐linked PEGDA/VP network and by comparing these spectra with those of the physical mixture and uncured resin.

### 4.6 Thermogravimetric Analysis of Resin and MNs

TGA was employed to assess thermal stability and to corroborate photopolymerization of the PEGDA/VP matrices by comparing the thermal events of individual components with those of the placebo MNs and API‐loaded patches. Printing was performed at ambient room temperature to avoid thermally induced degradation of the materials. Consequently, degradation temperatures were used as diagnostic indicators of polymer network formation (e.g. shifts in onset temperature, altered multi‐step mass‐loss profiles, and changes in residual mass/yield). TGA measurements were conducted using a Q50 thermobalance (TA Instruments, New Castle, DE, USA). Samples were heated from 25 to 500 °C in open aluminum pans at 10 °C min^−^^1^ under nitrogen (50 mL min^−^^1^), and mass loss was recorded as a function of temperature. Thermograms for polymers, resin, placebo MNs, and API‐loaded patches were acquired and analyzed using universal Analysis software. Each specimen was tested in triplicate to ensure reproducibility, and the resulting curves were evaluated for residual mass at 500 °C.

### 4.7 Thermal Transitions of MNs by DSC

DSC was used to characterize the thermotropic behavior of MN patches and to verify API incorporation. Thermograms were acquired using a DSC 214 Polymer (NETZSCH GmbH & Co. Holding KG, Selb, Germany) equipped with an autosampler. Approximately 7–8 mg of the sample was weighed into standard aluminum pans and hermetically sealed; an empty aluminum pan served as the reference. Measurements were performed from 10 to 400 °C at a heating rate of 20 °C min^−^^1^ under a nitrogen purge (40 mL min^−^^1^), following a published protocol [12]. Each specimen was analyzed in triplicate, and the results were averaged. Phase transitions were assigned from the thermograms, with melting points taken as the temperatures at the maxima of the principal endothermic peaks; where discernible, glass‐transition temperatures were determined at the midpoint of the baseline shift. Evidence of API incorporation into the cross‐linked matrix was assessed by changes in thermal signatures relative to physical mixtures and uncured resin, including depression or disappearance of drug‐melting endotherms, and shifts in polymer transitions.

### 4.8 Microscopic Morphology and Dimensional Fidelity of MNs

Morphology and dimensions of placebo and API‐loaded MNs patches were assessed using a stereomicroscope (Leica Microsystems EZ4W, Leica Microsystems, Wetzlar, Germany). From the calibrated micrographs, the MN height (tip‐to‐base) and base width/diameter, as well as patch length, width, and thickness, were measured and compared with the nominal dimensions specified in the digital CAD model and those obtained on the 3D‐printed parts to evaluate geometric fidelity. The surface microstructure was examined using a dual‐beam focused‐ion‐beam scanning electron microscope, Hitachi SU5000 field‐emission scanning electron microscope (Hitachi High‐Tech, Tokyo, Japan) equipped with EM Wizard software. For basic physical properties, the mean patch mass was determined on an analytical balance (Fisher Scientific) using *n* = 3 independent patches per formulation, and the results are reported as mean ± SD.

### 4.9 Insertion Performance on a Parafilm Skin Model

A ten‐layer Parafilm (Bemis Company Inc., Neenah, WI, USA) stack (individual sheets 10 × 10 cm; total thickness ≈1 mm) was used as a skin surrogate. MNs arrays were inserted using a Texture Analyzer (TA.XTplus, Stable Micro Systems, Surrey, UK) fitted with a cylindrical probe at a crosshead speed of 1.19 mm s^−^^1^, to a target load of 32 N, with a 30 s pause, following the published protocol [12]. After removal, the layers were separated, and perforations were counted under an optical microscope. The insertion efficiency was calculated as the percentage of needles that produced a perforation: insertion efficiency (%) = (number of holes created in Parafilm) / (total number of needles per patch) × 100. Pre‐ and post‐insertion MN heights were measured microscopically to quantify deformation (*n* = 3 arrays). In addition, manual (thumb‐press) insertion was assessed by pressing each patch onto the ten‐layer Parafilm stack for 30 s; perforations and MN heights before and after insertion were recorded using the same procedures.

### 4.10 Ex Vivo Penetration into Soft and Skin‐Like Tissues

*Ex vivo* insertion on soft‐tissue and skin surrogates. Ex vivo models were employed to approximate soft‐tissue and cutaneous conditions for MNs insertion testing, similar to the protocol from [26]. Fresh chicken breast (pectoralis major) was procured from a local retailer on the day of use, and neonatal porcine skin was obtained from a licensed slaughterhouse in accordance with local regulations; no live animals were used. On receipt, the skin was rinsed with distilled water, stored at ‐80 °C, and thawed at room temperature immediately prior to testing. Before experiments, the skin was thawed at room temperature and equilibrated in phosphate‐buffered saline to restore hydration. Although freezing and thawing may slightly affect mechanical properties such as elasticity and barrier integrity, frozen porcine skin is widely used as an ex vivo model for evaluating MN insertion and penetration behavior. The present study focuses on comparative insertion performance rather than direct equivalence to fresh in vivo tissue. For mounting, the tissue was placed on a clean Petri dish; for skin, hair was shaved with a disposable blade, the surface was gently blotted, and the specimen was positioned dermis‐side down on a glass slide. The edges were secured using laboratory tape to prevent lateral movement during application. Chicken breast muscle was selected as a soft‐tissue analogue because it is hairless, homogeneous, inexpensive, and readily available, offering consistent viscoelastic resistance for benchmarking mechanical insertion and detecting tip damage while avoiding confounding effects of the stratum corneum. Neonatal porcine skin complements this by better recapitulating the human cutaneous barrier and microchannel formation. MN patches were manually applied using thumb pressure for 30 s and then removed. The tissue surface was imaged immediately before and after application using an optical microscope under identical illumination and magnification settings. Image analysis was performed on post‐application micrographs to quantify insertion success (percentage of needles producing a visible microchannel) and microchannel size. Each condition was tested with *n* = 5 independent MN arrays, and the results are reported as mean ± SD.

### 4.11 Swelling and Degradation Behavior of MN Arrays

The swelling behavior of intact dissolving MN patches was evaluated in phosphate‐buffered saline (PBS, pH 7.4) at 32 ± 0.5 °C. Each patch was accurately weighed to obtain the initial dry mass (m_0_), immersed in 10 mL PBS, and at predefined time points (0.5, 1, 2, 4, 6, 8, 24, 48, 72 h) removed, gently blotted with lint‐free tissue to remove surface liquid, weighed (wet mass, m_t_), and returned to the same vial to continue the time‐course. Fluid uptake (%) was calculated in‐line as (m_t_ – m_0_) / m_0_ × 100. Measurements were performed on *n* = 3 independent patches per formulation and are reported as the mean ± SD. Polymer matrix degradation was assessed under the same conditions (PBS, pH 7.4, and 32 ± 0.5 °C). Patches were weighed to obtain m_0_, incubated in 10 mL PBS, and at 0.5, 1, 2, 4, 6, 8, 24, 48, 72 h removed, gently blotted, dried, and re‐weighed to determine the residual mass (m_t_). The mass‐loss (% degradation) percentage was computed as (m_0_ ‐m_t_) / m_0_ × 100. Each condition was tested in triplicate and summarized as the mean ± SD.

### 4.12 HPLC Quantification of Curcumin and 5‐Fluorouracil

Quantitative analysis of CUR and 5‐FU was performed on a HPLC system (1220 Infinity LC, Agilent Technologies, CA, USA) equipped with a UV detector. Separation was achieved using a C18 column (250 mm × 4.6 mm, Thermo Scientific, MA, USA) under reverse‐phase conditions. The analytical procedures were adapted from previously reported methods for CUR and 5‐FU quantification [35, 59], with optimization of the mobile phase composition, flow rate, and injection volume to suit the current formulations. For CUR, detection was performed at 425 nm, with the column maintained at 40 °C. The mobile phase consisted of ACN and 3% v/v acetic acid in water (58:42, v/v), delivered at 0.8 mL min^−^^1^. The injection volume was 10 µL, and the total run time was 9 min. Quantification was performed by external calibration (peak area) with sample identity verified by retention‐time concordance with authentic standards. For 5‐FU, detection was performed at 271 nm with the column held at 30 °C. The mobile phase comprised methanol: water (20:80, v/v) at a flow rate of 0.8 mL min^−^^1^. The injection volume was 20 µL, and the run time was 10 min. Quantification employed external calibration using peak‐area integration, and compound identity was confirmed by retention time matching to standards. Calibration curves for both analytes were linear (*R* ^2^ > 0.999) over the tested concentration range, and system suitability (retention‐time reproducibility and peak symmetry) was confirmed before analysis. The modified conditions yielded sharp, well‐resolved peaks and enabled precise, selective quantification of CUR and 5‐FU within the DLP‐printed dissolving MN formulations.

### 4.13 In Vitro Release and % Drug Content

Drug release was profiled under sink conditions at 32.0 ± 0.5 °C using a static diffusion set‐up via the bottle dissolution method. The MN patch was placed into a dialysis bag (cellulose membrane, molecular weight cut‐off 12–14 kDa) containing 1 mL of dissolution medium, after which the sealed bag was transferred into vials containing the remaining 9 mL of medium for the release study. Dialysis bags composed of cellulose membrane were activated before use by boiling in deionized water and subsequently rinsed thoroughly with fresh water. To accommodate the differing solubilities of the APIs, CUR release was examined in PBS (pH 6.5) containing 0.5% v/v Tween 80, a non‐ionic surfactant commonly employed to maintain sink conditions for poorly soluble compounds, such as CUR [35], whereas 5‐FU was tested in PBS (pH 7.4). Individual MN patches were immersed in 10 mL of their respective media, and aliquots were withdrawn at 0.5, 1, 2, 4, 6, 8, 24, 48, and 72 h; an equal volume of pre‐warmed fresh medium was immediately replaced to maintain a constant volume and composition. Drug concentrations were quantified by HPLC using external calibration and retention time matching to authentic standards under the drug‐specific UV conditions described above. Cumulative release (%) at the time it was calculated. Data are expressed as mean ± SD (*n* = 3 independent patches per time point). Cumulative release profiles were fitted to standard kinetic models, and goodness‐of‐fit metrics guided mechanistic interpretation. For the assay, MN patches (*n* = 3 per formulation) were finely powdered. CUR extracts were prepared with 10 mL of methanol, and 5‐FU extracts were prepared with 10 mL of water. Dispersions were vortex‐mixed, centrifuged, and the supernatants filtered (0.22 µm; PTFE for methanolic CUR and nylon for aqueous 5‐FU) before HPLC analysis. The drug assay (%) was calculated, and the results are presented as mean ± SD.

### 4.14 Stability Testing of Resin and MNs

Pragmatic stability testing was performed on API‐loaded PEGDA‐VP photopolymer resin and on printed MN arrays. Resin aliquots were stored in sealed, light‐protected containers in freeze under conditions (4 °C) and at room temperature (20–25 °C). MN arrays were stored under ambient laboratory conditions (uncontrolled humidity/light) and under temperature‐stress exposure (37 °C, without humidity control). At predetermined timepoints (1, 15, 30, 45, and 60 days), resins were visually inspected for color change, precipitation/phase separation, and handling changes; printability was screened using a standardized test print, with subsequent assessment of dimensional fidelity and residual uncured resin (surface tackiness/incomplete washing). MN arrays were examined for warping and tip/baseline defects, and dimensional fidelity was assessed by macroscopy and microscopy; mechanical integrity was evaluated using compression and insertion testing using parafilm. Drug content was quantified at each timepoint following extraction using HPLC. Because full ICH‐compliant (International Council for Harmonization) long‐term/accelerated protocols were not feasible, the study was positioned as a pragmatic screening assessment informed by ICH Q1A(R2) and photostability considerations (ICH Q1B) [60], consistent with stability approaches reported for PEGDA‐based drug photopolymers [61] and drug‐loaded MN patches [57].

### 4.15 Statistical Analysis

All quantitative results were evaluated using appropriate statistical tests and are presented as mean ± standard deviation (SD) where applicable. Statistical comparisons were performed using one‐way ANOVA or unpaired t‐tests in GraphPad Prism (GraphPad Software, Inc., USA). A 95% confidence level was applied throughout the analysis unless stated otherwise. Levels of statistical significance were denoted as follows: *p* ≤ 0.05, *p* ≤ 0.01, and *p* ≤ 0.001.

## Author Contributions

Data curation, formal analysis, Investigation, Software, Writing (original draft), writing (review), and editing were performed by [**Rutuja N. Meshram**]. Methodology, Validation, Writing – review & editing were performed by [**Dimitrios A. Lamprou**]. All authors read and approved of the final manuscript.

## Funding

This work was supported by the Joint Commissioner (Education Branch), Social Welfare – Maharashtra, India (Reference No. CWS/Edu/F.S./2023‐24).

## Ethical Statement

No animals were sacrificed specifically for this study. According to the institutional guidelines of Queen's University Belfast, the use of slaughterhouse‐derived tissues does not require formal ethical approval.

## Conflicts of Interest

The authors declare no conflicts of interest.

## Data Availability

The datasets generated and/or analyzed during the current study are available from the corresponding author upon reasonable request.

## ENDNOTE

1 Note: Color differences were used solely to distinguish formulations, and dyes were applied to enhance visibility of insertion sites.
